# *Drosophila* model of anti-retroviral therapy induced peripheral neuropathy and nociceptive hypersensitivity

**DOI:** 10.1242/bio.054635

**Published:** 2021-01-27

**Authors:** Keegan M. Bush, Kara R. Barber, Jade A. Martinez, Shao-Jun Tang, Yogesh P. Wairkar

**Affiliations:** 1 Neuroscience Graduate Program, University of. Texas Medical Branch, Galveston, TX 77555, USA; 2 Mitchell Center for Neurodegenerative Diseases, Department of Neurology, University of Texas Medical Branch, Galveston, TX 77555, USA; 3 Department of Neuroscience, Cell Biology, and Anatomy, University of Texas Medical Branch, Galveston, TX 77555, USA

**Keywords:** Dendrites, HIV, NRTI, Stability, Synapse

## Abstract

The success of antiretroviral therapy (ART) has improved the survival of HIV-infected patients significantly. However, significant numbers of patients on ART whose HIV disease is well controlled show peripheral sensory neuropathy (PSN), suggesting that ART may cause PSN. Although the nucleoside reverse transcriptase inhibitors (NRTIs), one of the vital components of ART, are thought to contribute to PSN, the mechanisms underlying the PSN induced by NRTIs are unclear. In this study, we developed a *Drosophila* model of NRTI-induced PSN that recapitulates the salient features observed in patients undergoing ART: PSN and nociceptive hypersensitivity. Furthermore, our data demonstrate that pathways known to suppress PSN induced by chemotherapeutic drugs are ineffective in suppressing the PSN or nociception induced by NRTIs. Instead, we found that increased dynamics of a peripheral sensory neuron may possibly underlie NRTI-induced PSN and nociception. Our model provides a solid platform in which to investigate further mechanisms of ART-induced PSN and nociceptive hypersensitivity.

This article has an associated First Person interview with the first author of the paper.

## Abstract (precis)

**Summary:** Nucleoside reverse transcriptase inhibitors (NRTIs) that are important components of anti-retroviral therapies also cause peripheral sensory neuropathies (PSN). This article investigates ways in which NRTIs may cause PSN and outlines ways to better understand the mechanisms underlying it.

## INTRODUCTION

While the antiretroviral therapy (ART) has been successful in controlling the symptoms in acquired immunodeficiency syndrome (AIDS) patients, a significant proportion of patients on ART develop peripheral sensory neuropathy (PSN) (Sacktor, 2002; Hulgan et al., 2005; Lichtenstein et al., 2005; Kallianpur et al., 2006; Nakamoto et al., 2010; Evans et al., 2011; Schutz and Robinson-Papp, 2013). These symptoms include pain and/or numbness in the extremities (Dubinsky et al., 1989). Thus, while NRTIs can control the HIV infection, they can also cause neurological complications (Berger et al., 1993; Breen et al., 2000; Dalakas, 2001; Dalakas et al., 2001; Cohen, 2002; Rasmussen et al., 2018). Therefore, chronic administration of ART may also contribute to the neurological complications, including PSN (Cupler and Dalakas, 1995; Dalakas, 2001; Dalakas et al., 2001; Ellis et al., 2010; Robertson et al., 2010; Luma et al., 2012). In particular, nucleoside reverse transcriptase inhibitors (NRTI), the backbone of ART regimens, are especially relevant in this regard because their neurotoxicity is well established (Cupler and Dalakas, 1995; Wulff et al., 2000; Reliquet et al., 2001; Keswani et al., 2006; Margolis et al., 2014; Wu et al., 2017). For example, d4T, an NRTI still used in some resource-limited countries, has shown to be neurotoxic not only in HIV-patients but even in uninfected subjects that received it as prophylaxis (Owino et al., 2013). However, little is known about the mechanisms underlying the neurotoxicity of NRTIs in peripheral nerves. Previous studies have suggested various mechanisms of neurotoxicity, including mitochondrial damage (Lewis and Dalakas, 1995; Lewis et al., 2005; Lin et al., 2009) and neuroinflammation (Sanchez and Kaul, 2017; Wu et al., 2017; Yuan et al., 2018). Thus, the current research on the neurotoxicity of NRTIs is focused on describing their detrimental impacts on specific biological pathways such as mitochondrial homeostasis and neuronal apoptosis. While these approaches have been helpful, the mechanistic insights into the effects of NTRI-regulated biological pathways to the PSN have not been conclusively established. We have taken advantage of the similarity between *Drosophila* and mammalian sensory neurons (Caldwell and Tracey, 2010; Im and Galko, 2012; Milinkeviciute et al., 2012) to establish a novel strategy that will allow for genome-wide unbiased forward genetic screens to understand the molecular mechanisms that play key roles in regulating the development of NRTI-induced neurotoxicity in the peripheral nerves. Interestingly, when *Drosophila* larvae are subjected to NRTI treatment, the peripheral branches of sensory neuron dendrites show an increased instability and fragmentation-like phenotype as compared to the untreated larvae. Moreover, genetically restoring stability to the dendrites of the peripheral sensory neurons significantly suppresses their degeneration. In addition to the fragmentation-like phenotype in the sensory neurons, the larvae where the sensory neurons are genetically stabilized also show a significant reduction in nociceptive hypersensitivity, indicating that the instability of peripheral sensory neurons may possibly drive the degeneration and the nociceptive hypersensitivity in the *Drosophila* model. Thus, our study provides a genetically amenable platform to further dissect the molecular pathways underlying NRTI-induced PSN and nociceptive hypersensitivity.

## RESULTS

### Exposure to *AZT* induces thermal and mechanosensory nociceptive hypersensitivity in *Drosophila*

**Fig. 1.**
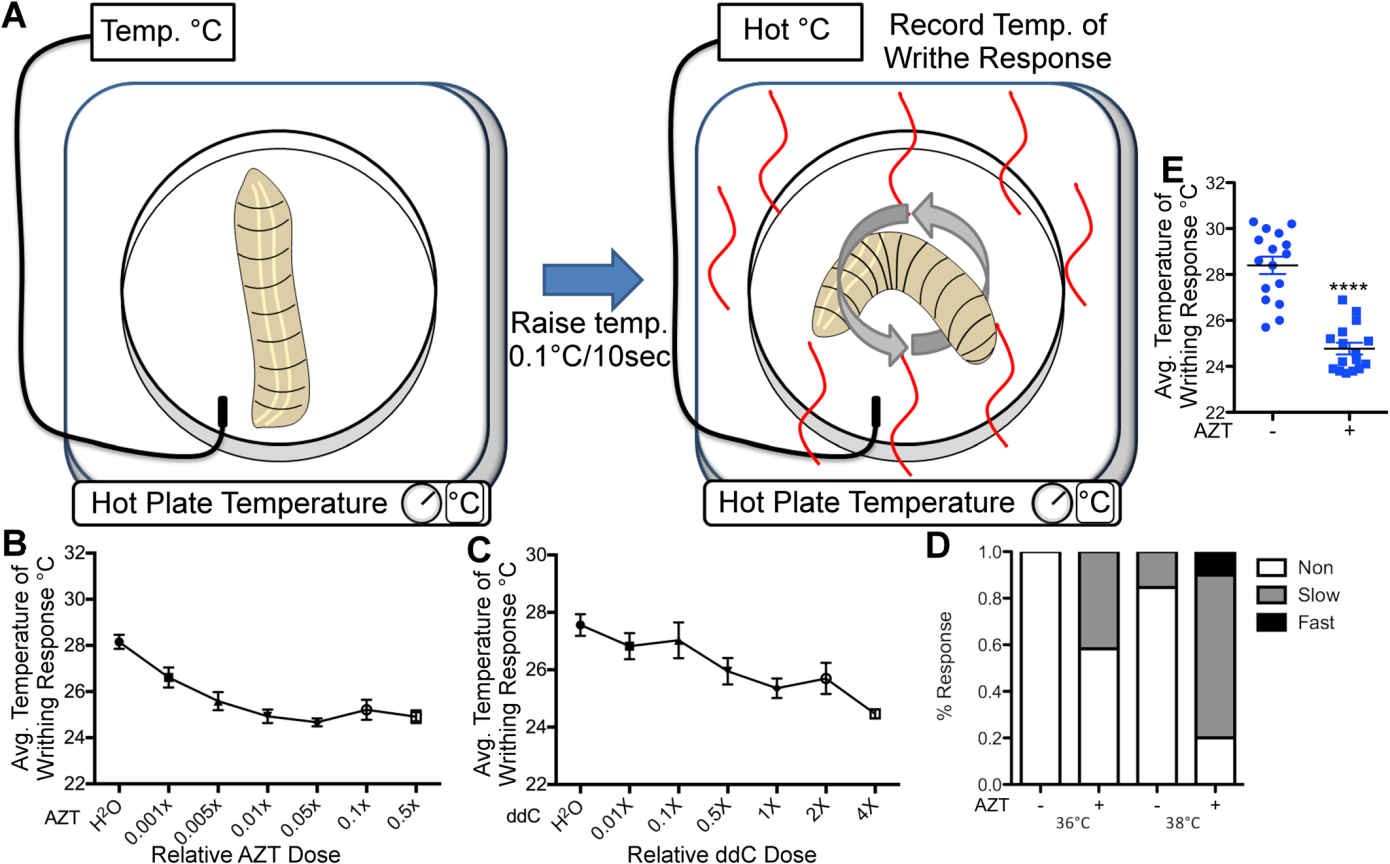
**NRTIs induce nociceptive hypersensitivity in *Drosophila*.** (A) Experimental set up for larval thermal nociception assay. Larvae were subjected to small increases in temperatures and monitored for writhing response. Temperature of writhing response was recorded using a sensitive probe. (B) Quantification of thermal nociception response of larvae to increasing doses of AZT, mean with s.e.m. A total of 16 larva were used per dose with three repetitions. *F*(6, 105)=14.3, *P*=7.68E-12, one-way ANOVA. (C) Quantification of writhing response to increasing doses of ddC, mean with s.e.m. eight larva were used per dose with three repetitions each. *F*(6, 49)=6.78, *P*=2.82E-5, one-way ANOVA. (D) Quantification for the thermal nociception response to thermal probe at 36°C and 38°C. Larvae raised on AZT exhibit significantly more nociceptive response at both temperatures. Slow response: larvae responding between 15–20 s; Fast response: larvae responding immediately to 5 s after thermal probe application. (E) Quantification of nociception temperature for WT larva in normal and 0.26 µg/ml (0.01X) AZT-containing food. *P*=5.02E-7, *t*-test. ****P<0.0001; error bars=s.e.m.

*Drosophila* larval model has been previously used in understanding the mechanisms of nociception (Caldwell and Tracey, 2010; Lesch et al., 2010; Neely et al., 2010; Im and Galko, 2012; Milinkeviciute et al., 2012; Khuong and Neely, 2013). When subjected to noxious stimuli, like high temperatures, the larvae respond by a characteristic ‘corkscrew-like’ escape behavior, also known as writhe (Yoshino et al., 2017), which has been successfully exploited to screen for genes involved in nociception (Caldwell and Tracey, 2010; Neely et al., 2010; Zhong et al., 2010; Honjo et al., 2016). Larvae that are sensitive to these noxious stimuli generally respond with writhe at a lower threshold than the control larvae. We adopted this established behavioral paradigm to test whether exposure to NRTIs can induce nociceptive hypersensitivity in wild-type (WT) larvae. We used a water bath made of polypropylene fitted with a sensitive temperature-measuring probe that can detect temperature fluctuations of 0.1°C (Fig. 1A and Movie 3). To test for nociceptive hypersensitivity, the temperature of the water bath was ramped up gradually in 0.1°C/10 s increments. A camera attached to the microscope tracked both the rise in temperature and larval movements (Fig. 1A). A writhing response by the larvae was recorded as a nociceptive hypersensitive response if the larvae showed at least three corkscrew-like movements without a stop at a temperature that was lower than the one that induced a similar reaction in WT larvae. First, we sought to optimize the dosage of NRTIs for *Drosophila* larvae. For this, we used a human equivalent dose of two NRTIs: AZT (Zidovudine or Azidothymidine) and ddC (Zalcitabine). Using a recent study that has used drugs mixed in the food to feed larvae (Bhattacharya et al., 2012), we estimated that 26 µg/ml food volume of AZT and 0.14 µg/ml food volume of ddC would be an ideal starting point (see Materials and Methods for details). Although this dose induced thermal hypersensitivity in the larvae it also induced a significant amount of lethality (30% in AZT and >80% in ddC, *n*=20, *P*<0.01). Therefore, we used two dilutions of this concentration (0.1×, and 0.05×) to establish an optimal dose that would allow us to test nociceptive hypersensitivity without causing significant lethality.

WT flies were transferred into vials that had drugs (AZT or ddC) mixed into the food. Control flies were transferred on the same day at the exact same time onto food prepared identically, except for the addition of drugs (AZT−/ddC−). Flies were allowed to lay eggs in AZT, ddC, or AZT−/ddC− food for about 3 days before being discarded. Wandering third instar larvae were collected and used for the nociceptive hypersensitivity experiments. We found that larvae raised on AZT or ddC food showed significantly lower temperature thresholds for the nociceptive writhe (at least 3°C) as compared to the control flies raised on AZT−/ddC− food (Fig. 1B,C, and Movie 3). This is a significant change given that the thermosensory neurons in flies can sense minute temperature differences (Babcock et al., 2009; Gallio et al., 2011). The minimum concentration of drug needed to decrease the temperature to see an observable writhing response with AZT was only 1/100th the estimated dosage at 0.26 µg/ml and did not induce lethality in the larvae (Fig. S1). However, even the minimum concentration of ddC (0.14 µg/ml) required to induce a significant nociceptive hypersensitivity was toxic (50% lethality, *n*=20, *P*<0.0001) making AZT a preferred drug to test the effects of NRTI induced neuropathy.

To confirm our data, we used the thermal probe method to test thermal nociception (Tracey et al., 2003; Chattopadhyay et al., 2012). This method relies on the consistently observable writhing response of larvae when the larvae are touched using a heated probe. This method has two advantages: first, it involves localized heat application as opposed to the global temperature increases encountered by larvae in a water bath; second, this method allows one to reliably test rapid response to thermal stimulation as opposed to the slow response generated using a temperature bath. When a thermal probe heated to 36°C was touched to the WT larvae raised in AZT^−^/ddC^−^, they did not show a significant response. However, the AZT/ddC^−^ larvae start showing a writhing response when the probe is heated to 38°C. Interestingly, 40% of larvae raised on AZT containing food showed a writhing response even at 36°C (Fig. 1D). While 15% of AZT^−^/ddC^−^ larvae also showed a writhing response at 38°C, this response was exaggerated in (80%) larvae raised on AZT at 38°C. Moreover, 15% of them showed a very fast response (Fig. 1D). While these data demonstrate that exposure to AZT induces nociceptive hypersensitivity, we wanted to test whether these responses were manifested by the C4da neurons that are responsible for heat sensitivity in *Drosophila* (Tracey et al., 2003; Hwang et al., 2007). To test this, we expressed the tetanus toxin light chain (UAS-TeTxLC) in C4da neurons using ppk-Gal4, which specifically silences these neurons (Ainsley et al., 2003). As expected, flies expressing TeTxLC showed no response to temperature changes in either AZT/ddC^−^ larvae or larvae raised on AZT, indicating that C4da neurons largely drive the thermal nociceptive hypersensitivity response of NRTIs (Fig. S3A). Finally, as newer NRTIs are introduced regularly, we wanted to test whether these newer NRTIs also induce nociceptive hypersensitivity. Therefore, we performed the same assays with newer NRTIs-Emtricitabine (FTC), Abacavir (Babcock et al., 2009), and Tenofovir (Tenofovir) (Fig. S4A). All the newer NRTIs tested showed increased nociceptive hypersensitivity to thermal stimulation, indicating that most NRTIs induce nociceptive hypersensitivity in the *Drosophila* model.

**Fig. 2.**
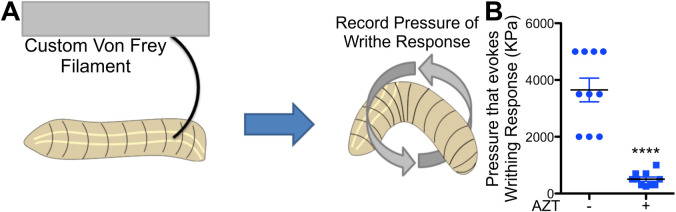
**AZT also induces mechanical allodynia.** (A) Diagram of the Von Frey mechanical nociception test for *Drosophila* larva. A set of calibrated Von Frey filaments with increasing pressure from 100 kPa–5000 kPa were used to stimulate dorsal A6 or A7 segment of larva. Larvae were monitored for 30 s for nociceptive behavior before continuing to the next filament. (B) Quantification of sensitivity to mechanical stimulation in WT larva in normal and 0.26 µg/ml (0.01X) AZT-containing food. *P*=1.56E-5, *t*-test. ****P<0.0001; error bars=s.e.m.

Since anti-retroviral therapy can also lead to the development of mechanical allodynia (Huang et al., 2014; Yuan et al., 2018), we asked whether the larvae exposed to NRTI also showed nociceptive hypersensitivity to mechanical stimuli. To perform these assays, we designed and calibrated Von Frey filaments, in house. Von Frey filaments were calibrated for specific pressures (described in Materials and Methods) and consistently applied to the posterior third of the larvae (Fig. 2A). Von Frey filaments induced nociceptive writhe in larvae raised on AZT at lower pressures compared to larvae raised on AZT− food, suggesting that exposure to AZT also lowers the threshold to mechanical stimulation (Fig. 2B). Similar results were also obtained using newer NRTIs (Fig. S4B). Like thermal nociception, we also tested whether the response to AZT was dependent on C4da neurons. As expected, most larvae raised on AZT did not respond when TeTxLC was driven in C4da neurons. However, 30% of WT larvae responded when subjected to higher pressure (Fig. S3B). As a control, we also tested whether there were any issues with general motility in larvae raised on NRTIs using the locomotor assay (Nichols et al., 2012) and did not find any significant defects in the motility of the larvae (Fig. S2A). We also tested for any defects in the development of the larval musculature and found that the size of the muscles did not differ significantly between the larvae raised on AZT/ddC^−^ food and those raised on AZT (Fig. S2B). Finally, the larvae raised on AZT eclosed from pupa at that same time as the WT larvae raised in AZT− food (Data not shown). Together, these data demonstrate that larvae exposed to NRTIs show both thermal and mechanical nociceptive hypersensitivity.

### Exposure to AZT shows signs of degeneration in sensory neurons

**Fig. 3.**
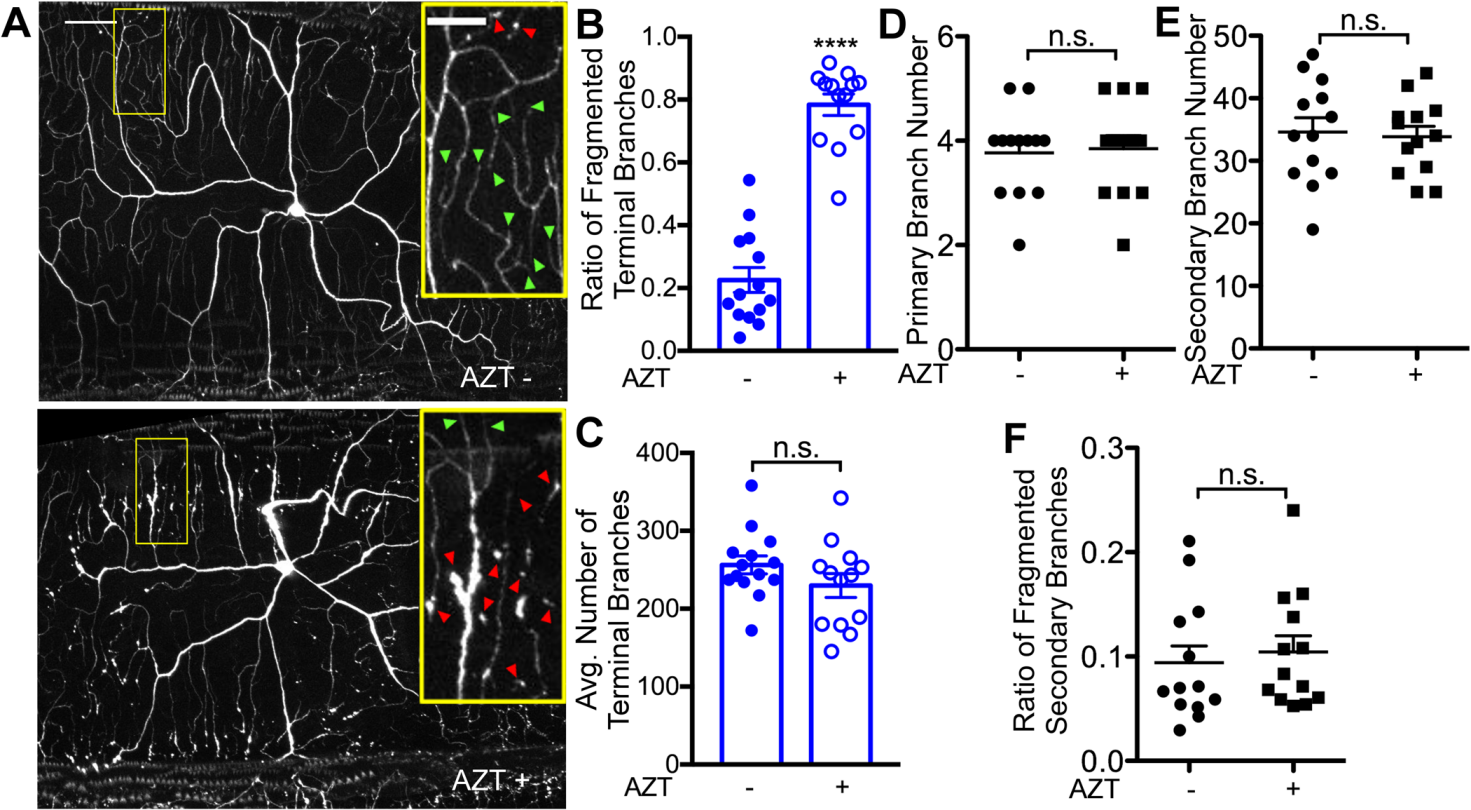
**Exposure to AZT leads to fragmentation of distal dendrites.** (A) Representative images of the C4da sensory neurons of third instar larvae comparing vehicle and AZT-dosed larva. Inset arrows identify terminal branches that are intact (green) and fragmented (red). (B) The quantity of terminal branches of C4da sensory neurons is unaffected by AZT. *P*=0.177, *t*-test. (C) The proportion of C4da terminal branches that exhibit fragmentation is increased by AZT exposure. *P*=9.62E-11, *t*-test. (D) The quantity of primary branches of C4da sensory neurons in unaffected by AZT. *P*=0.823, *t*-test. There was no detectable fragmentation of C4da primary branches in control or AZT dosed larva. (E) The quantity of secondary branches of C4da sensory neurons in unaffected by AZT. *P*=0.788, *t*-test. (F) The proportion of C4da secondary branches that exhibit fragmentation is unaffected by AZT exposure. *P*=0.645, *t*-test. Refer to Fig. S6 for sensory neuron response to Taxol. Vehicle −; AZT +. ****P<0.0001; error bars=s.e.m.; scale bar=50 µm; inset scale bar=20 µm.

Skin biopsies of patients with painful neuropathy (or distal sensory neuropathy, DSP) show degeneration of peripheral sensory neurons (Polydefkis et al., 2002; Polydefkis, 2006; Obermann et al., 2008; Phillips et al., 2014). To test whether the *Drosophila* model shows signs of peripheral sensory neuron degeneration, we assessed the sensory neurons in larvae exposed to AZT. To test this, we used the ppk-EGFP^5^ line that expresses EGFP under pickpocket promoter and labels the Class 4 da neurons (C4da) (Grueber et al., 2003). These neurons are necessary for nociception induced by noxious temperature and mechanical stimuli (Hwang et al., 2007; Zhong et al., 2010). Quantification of C4da neuron terminal dendrite branch number and proportion of branches that exhibited possible fragmentation (discontinuous GFP fluorescence) revealed that the most obvious difference observed between larvae raised on AZT− media versus those raised on AZT had a significant increase in the proportion of possibly fragmented terminal (distal) dendrites (Fig. 3B), consistent with a decrease in nerve fiber density observed in HIV neuropathies (Polydefkis et al., 2002). Notably, larvae raised on AZT did not have a significant change in the number of terminal dendrites (Fig. 3C), or the primary and the secondary branches of the sensory neurons (Fig. 3D,E). Also, the primary and secondary branches did not show fragmentation phenotype (Fig. 3F). Finally, FTC-a newer NRTI also showed fragmentation similar to that of AZT (Fig. S5E). Next, we tested whether the possible terminal dendrite fragmentation caused by AZT was similar to the one that has been reported for Taxol, a chemotherapeutic drug (Bhattacharya et al., 2012). Consistent with the previous report, we found a significant fragmentation of terminal dendrites in larvae exposed to Taxol. However, notably, the fragmentation induced by Taxol was much more severe than that induced by AZT (Fig. S6).

While our method of dissection (see Materials and Methods) did not produce fragmentation of terminal dendrites (up to 15 min), we wanted to be careful that the dissection procedure was not causing the fragmentation. For this reason, we took advantage of the fact that EGFP fluorescence can be visualized via the cuticle of live larvae. Therefore, we performed live imaging of ppk-EGFP^5^ lines that were exposed to AZT and those that were not exposed to AZT. Similar to what we found in our dissected preparations; live imaging of larvae also showed significant fragmentation-like phenotype in the terminal dendrites in flies exposed to AZT as compared to those that were not exposed to AZT (Fig. S8). Previous report on flies that were exposed to Taxol suggest that peripheral sensory neurons can also show the disruption of microtubule networks (Brazill et al., 2018). To test whether microtubules were disrupted in flies exposed to AZT, we stained control flies to flies exposed to AZT with anti-Futsch antibody that stains only the neuronal microtubules (Hummel et al., 2000). Futsch staining was clearly disrupted in the terminal dendrites; similar to the EGFP fluorescence, indicating that treatment with AZT can also lead to the disruption of microtubules in the dendrites (Fig. S9C). Together, these data indicate that exposure of *Drosophila* larvae to AZT causes fragmentation-like phenotype of terminal dendrites.

### Fragmentation-like phenotype observed with AZT may not depend on *wnd*/dlk pathway

The data that both AZT and Taxol cause fragmentation-like phenotype in terminal dendrites suggested to us that the mechanisms that underlie the fragmentation might be similar. Work in *Drosophila* and cultured DRG neurons has shown that Taxol-induced axonal degeneration can be suppressed by downregulating the Wallenda/di-leucine zipper kinase (DLK) pathway (Miller et al., 2009; Bhattacharya et al., 2012). Interestingly, downregulating DLK also delays Wallerian degeneration, a form of axon degeneration caused due to the damage to axons (Miller et al., 2009; Shin et al., 2012; Xiong and Collins, 2012), suggesting that the DLK pathway might be one of the common pathways that mediate the injury response signaling. Furthermore, a recent study has also suggested that highwire, a ubiquitin ligase (DiAntonio et al., 2001), that functions upstream of *wnd*/dlk pathway (Collins et al., 2006), can mediate nociceptive hypersensitivity (Honjo and Tracey, 2018). Therefore, we wanted to test whether this pathway might play a role in mediating the response to AZT.

**Fig. 4.**
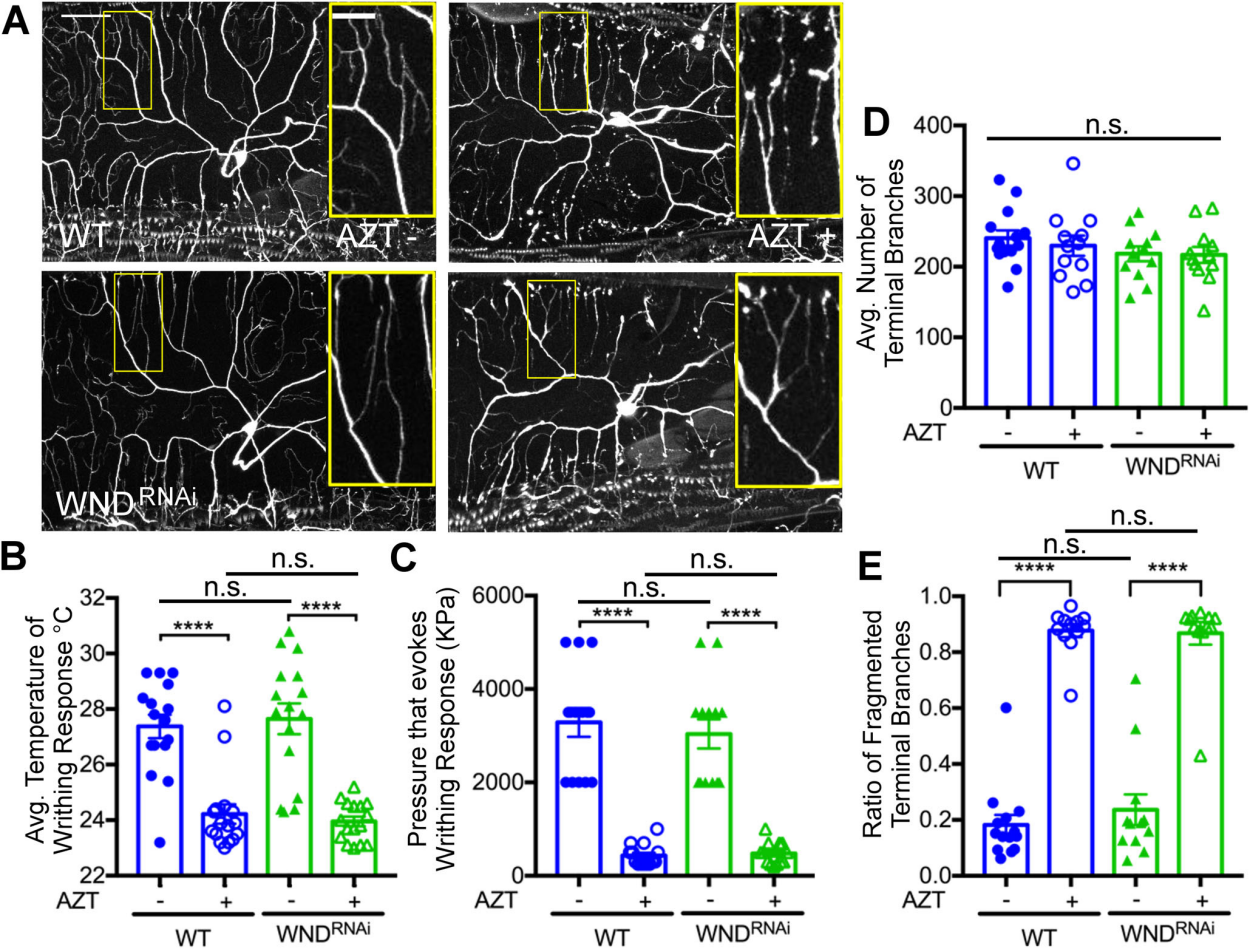
**NRTI induced fragmentation of terminal dendrites may not depend on *wnd*/DLK pathway.** (A) Representative images of the C4da sensory neurons of third instar larvae showing vehicle and AZT treated WT and *wnd*^RNAi^ larvae. (B) Quantification of temperature required to elicit nociceptive behavior in WT and *wnd*^RNAi^ larvae. *F*(3, 60)=25.0, *P*=1.26E-10, one-way ANOVA. Post hoc Bonferroni (WT, WT AZT) *P*=2.42E-6, (*wnd*^RNAi^, *wnd*^RNAi^ AZT) *P*=4.58E-7, (WT, *wnd*^RNAi^) *P*=0.693, (WT AZT, *wnd*^RNAi^ AZT) *P*=0.506. (C) Quantification of pressure needed to induce mechanical nociception in WT and *wnd*^RNAi^ larvae. *F*(3, 50)=48.9, *P*=6.60E-15, one-way ANOVA. Post hoc Bonferroni (WT, WT AZT) *P*=1.59E-9, (*wnd*^RNAi^, *wnd*^RNAi^ AZT) *P*=3.04E-8, (WT, *wnd*^RNAi^) *P*=0.579, (WT AZT, *wnd*^RNAi^ AZT) *P*=0.627. (D) Quantification of terminal branches of C4da sensory neurons in WT and *wnd*^RNAi^ larvae. *F*(3, 46)=0.930, *P*=0.434, one-way ANOVA. (E) Quantification of proportion of C4da terminal branches that exhibit fragmentation induced by AZT exposure in WT and *wnd*^RNAi^ larvae. *F*(3, 46)=92.8, *P*=1.57E-19, one-way ANOVA. Post hoc Bonferroni (WT, WT AZT) *P*=3.82E-14, (*wnd*^RNAi^, *wnd*^RNAi^ AZT) *P*=4.74E-9, (WT, *wnd*^RNAi^) *P*=0.405, (WT AZT, *wnd*^RNAi^ AZT) *P*=0.844. WT=blue and *wnd*^RNAi^ larvae=green. Vehicle (−); AZT (+). ****P<0.0001; error bars=s.e.m.; scale bar=50 µm; inset scale bar=20 µm.

**Fig. 5.**
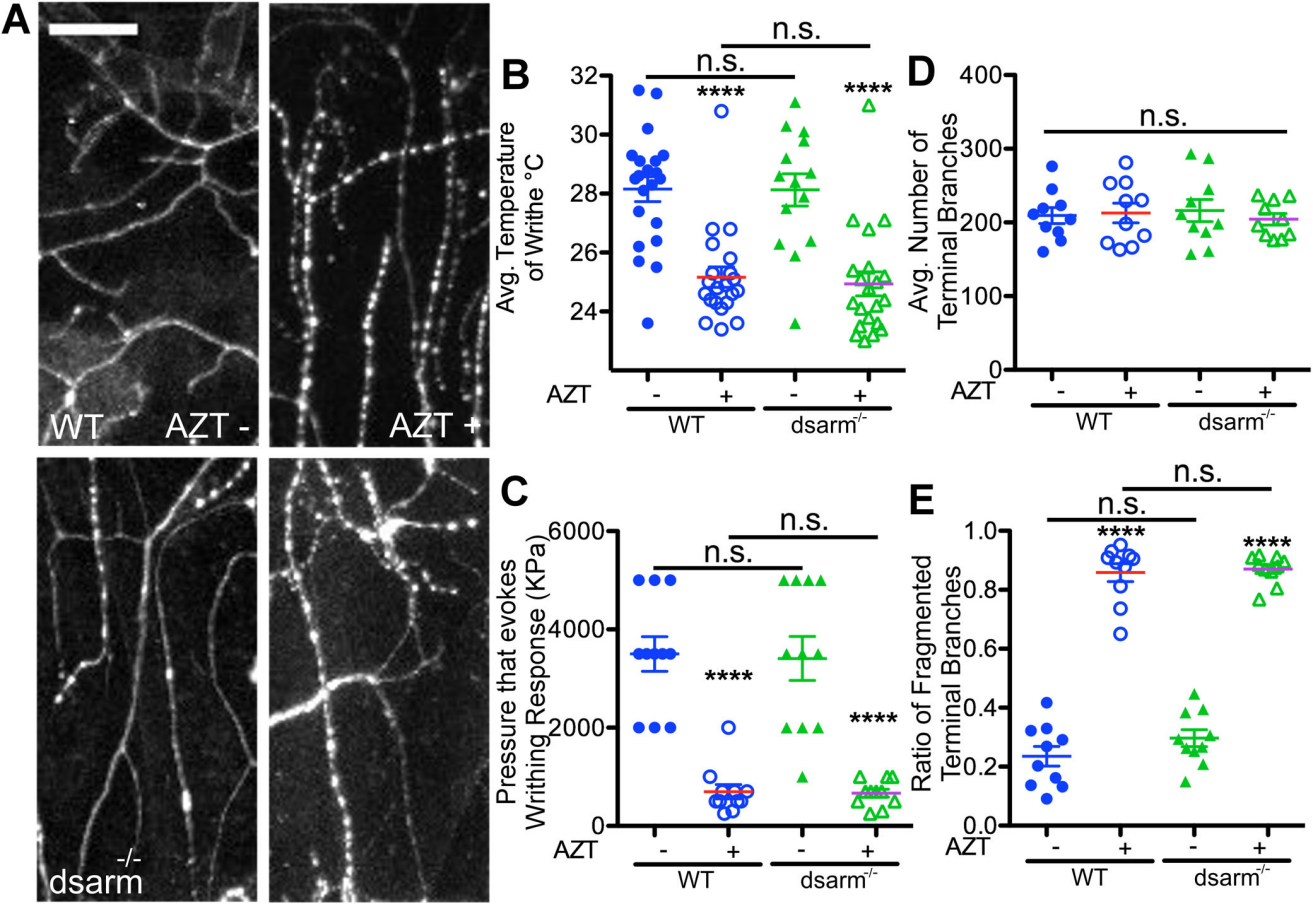
**Pathways underlying NRTI induced degeneration are likely distinct from those regulating chemotherapy induced degeneration,** (A) Representative images of the sensory neurons of third instar drosophila larvae comparing vehicle and AZT dosed WT and dsarm^−/−^ (Ect4^−/−^) larvae grown in vehicle or AZT-laced food. (B) AZT induces a significant decrease in the threshold of temperature stimulus required to elicit nociceptive behavior in both WT and dsarm^−/−^ larvae. *F*(3, 93)=20.1, *P*=3.87E-10, one-way ANOVA. Post hoc Bonferroni (WT, WT AZT) *P*=9.50E-8, (dsarm^−/−^, dsarm^−/−^ AZT) *P*=1.86E-5, (WT, dsarm^−/−^) *P*=0.641, (WT AZT, dsarm^−/−^ AZT) *P*=0.672. (C) AZT induces a significant decrease in the threshold of mechanical stimulus required to elicit nociceptive behavior in both WT and dsarm^−/−^ larvae. *F*(3, 40)=29.4, *P*=3.34E-10, one-way ANOVA. Post hoc Bonferroni (WT, WT AZT) *P*=3.81E-7, (dsarm^−/−^, dsarm^−/−^ AZT) *P*=6.56E-6, (WT, dsarm^−/−^) *P*=0.874, (WT AZT, dsarm^−/−^ AZT) *P*=0.871. (D) The quantity of terminal branches of sensory neurons is unaffected by AZT in both WT and dsarm^−/−^ larvae. *F*(3, 36)=0.173, *P*=0.914, one-way ANOVA. (E) The proportion of terminal branches that exhibit fragmentation is increased by AZT exposure in both WT and dsarm^−/−^ larvae. WT quantification in blue and dsarm^−/−^ larvae quantification in green. *F*(3, 36)=154, *P*=1.32E-20, one-way ANOVA. Post hoc Bonferroni (WT, WT AZT) *P*=5.61E-11, (dsarm^−/−^, dsarm^−/−^ AZT) *P*=7.90E-13, (WT, dsarm^−/−^) *P*=0.182, (WT AZT, dsarm^−/−^ AZT) *P*=0.724. Vehicle −; AZT+. N.S.=*P*>0.05, ****P<0.0001; error bars=s.e.m.; scale bar=20 µm.

To test this hypothesis, well-characterized RNAi lines of *wnd*/*dlk* were exposed to AZT along with WT controls (Xiong et al., 2010; Xiong and Collins, 2012; Valakh et al., 2013; Ma et al., 2015). If knockdown of *wnd*/dlk blocked the neurotoxicity induced by AZT, we expected to observe a suppression of fragmentation in *wnd*/dlk knockdown larvae raised on AZT. In contrast, knockdown of *wnd* showed an increase in the fragmentation-like phenotype of terminal dendrites, which was not significantly different from that induced by AZT in WT larvae (Fig. 4A,D,E). To further confirm these observations, we performed the same experiment on *dSarm* mutants. SARM (sterile a-motif-containing and armadillo-motif containing protein) also works in the Wallerian degeneration pathway and mutations in d*SARM* also suppress Wallerian degeneration (Osterloh et al., 2012; Conforti et al., 2014; Freeman, 2014). We found that mutations in *dsarm* were also ineffective in suppressing the fragmentation-like phenotype induced by AZT (Fig. 5A,D,E). These data indicate that unlike Taxol-induced fragmentation, the mechanisms that underlie the possible fragmentation of terminal dendrites in larvae exposed to AZT may not be mediated by the *wnd*/dlk or SARM pathway.

Although *wnd*/DLK knockdown (or d*SARM* mutants) did not suppress the fragmentation-like phenotype of dendrites, we asked whether these pathways might still be able to suppress the nociceptive hypersensitivity induced by the NRTIs without suppressing the fragmentation. However, knockdown of *wnd*/DLK (Fig. 4B,C) or mutations in d*Sarm* (Fig. 5B,C) did not suppress the nociceptive hypersensitivity induced by AZT, indicating that these pathways may not play a significant role in suppressing the neurotoxicity induced by NRTIs. Finally, we also performed general motility tests on d*Sarm* and *wnd*^RNAi^ lines and did not find any significant defects in the general motility of these larvae (data not shown).

### Exposure to AZT leads to an increase in dynamic dendrites

**Fig. 6.**
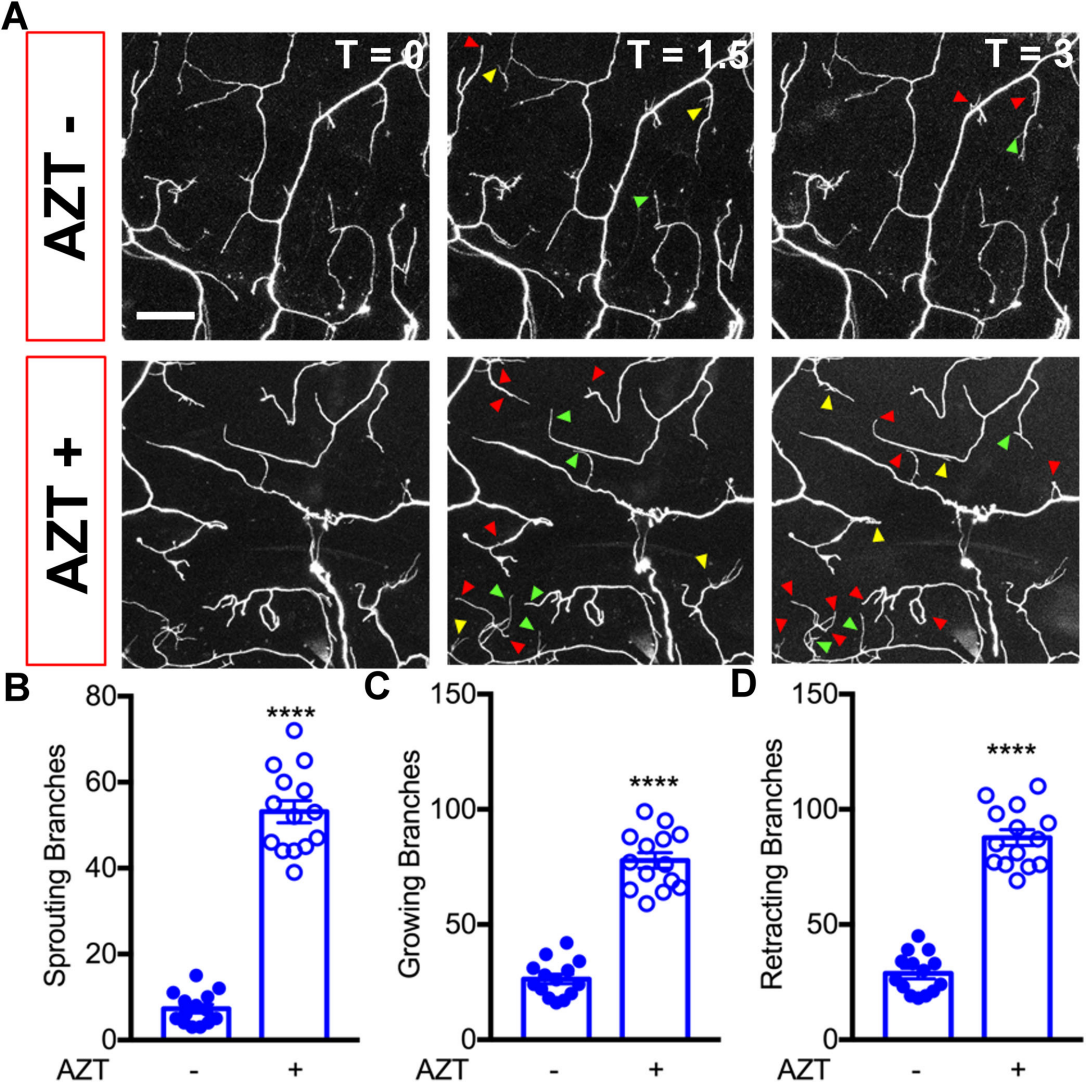
**NRTI exposure increases the number of dynamic dendrites.** (A) Representative images of C4da terminal dendrites imaged over a 3 h time period in larvae exposed to vehicle and AZT. Time points shown are: 0, 1.5 h, and 3 h. (B–D) Quantification of terminal dendrites showing sprouting (B) (*P*=2.34E-15, *t*-test), growing (C) (*P*=3.77E-13, *t*-test), and retraction (D) *P*=8.79E-14, *t*-test. Vehicle=(−); AZT=(+). ***P<0.001; error bars=s.e.m., scale bar=20 µm. Red arrowheads shows retracting dendrites, green arrow shows growing dendrites and yellow arrows show sprouting dendrites. Refer to Movies 4 and 5 for video examples of live imaging frames and dendrite changes. Refer to Fig. S7 for Taxol effects on sensory neuron dynamics.

To understand the cellular mechanisms that may underlie the fragmentation-like phenotype in terminal dendrites, we turned to time-lapse imaging studies. Previous studies have suggested that chronic instability of dendrites might be a precursor for neurodegeneration (reviewed in Koleske, 2013). Therefore, we wanted to test whether the terminal dendrites that showed the fragmentation were more dynamic in AZT exposed larvae. To test this, we performed time-lapse imaging on C4da neurons labeled by ppk-EGFP^5^ in live animals without anesthesia (see Materials and Methods for details). Images were acquired from the entire dendritic field of one to two C4da neurons in between body segments 2–5, every 10 min for 3 h. Random section of the image from each time point was compared to its previous time point to determine the number of branches that were extending, retracting, or sprouting. These experiments should test for any difference between the dynamic nature of the dendrites between the two experimental conditions (AZT+ and AZT−). These experiments revealed that the number of terminal dendrites that were exhibiting dynamic changes was significantly increased in larvae raised on AZT (Fig. 6, also see Movie 4, AZT−; Movie 5, AZT+). These data suggest that WT larvae that were not raised on AZT have more stable terminal dendrites than those exposed to AZT. Furthermore, larvae whose dendrites were exposed to AZT first extended and then retracted the dendrites similar to a smaller percentage of WT dendrites not exposed to AZT (Fig. 6 and Movie 4). In contrast, majority of the dendrites exposed to Taxol showed only retractions and had significantly fewer extending or sprouting dendrites (Fig. S7), further highlighting the possible underlying differences in the cellular mechanisms of fragmentation induced by AZT and Taxol. Based on these data, we conclude that exposure to AZT leads to more dynamic dendrites, which may make them vulnerable to fragmentation (Koleske, 2013).

### Increased expression of Par-1 suppresses AZT induced fragmentation-like phenotype and nociception

**Fig. 7.**
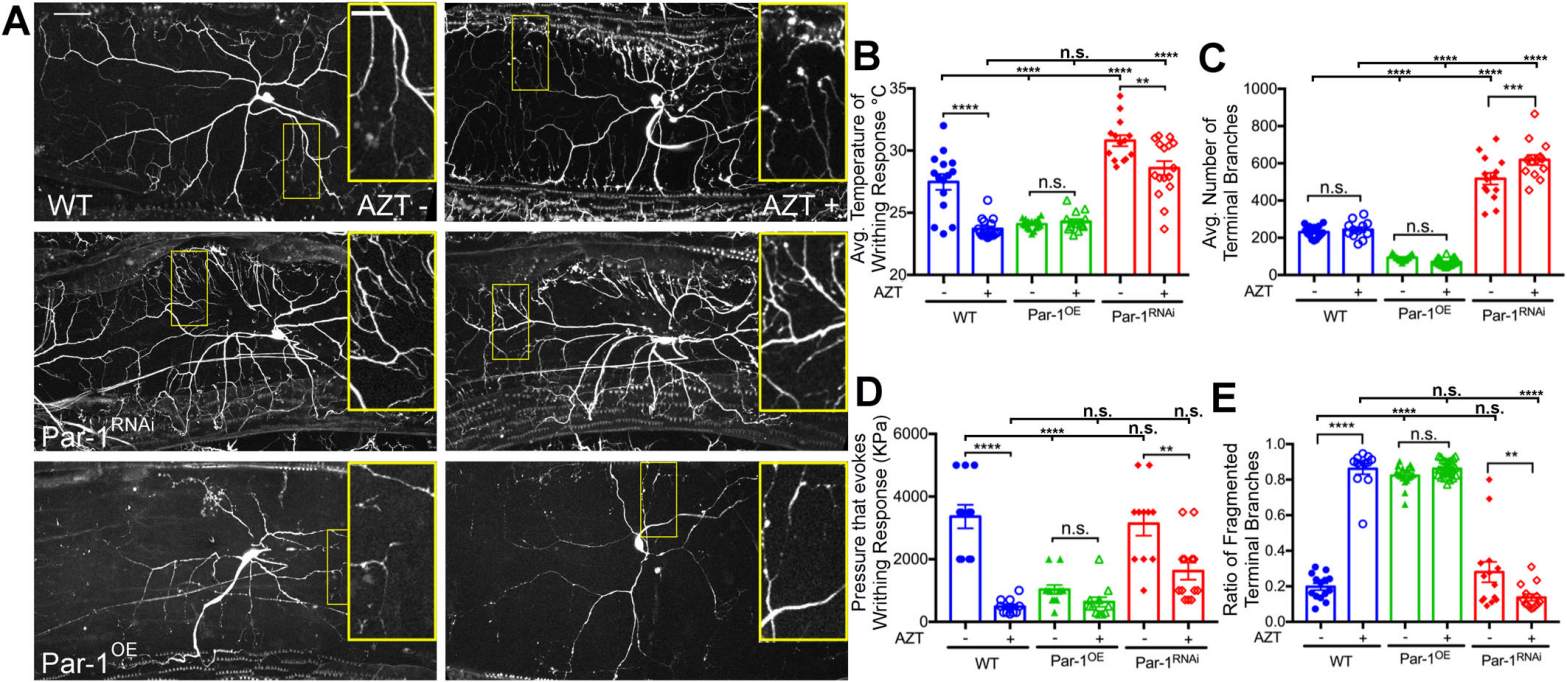
**Decrease in Par-1 suppresses fragmentation induced by AZT.** (A) Representative images of GFP labeled-C4da sensory neurons of third-instar larvae raised on food containing vehicle or AZT. The genotypes are indicated on the figure and are: WT, Par-1 overexpression (Par-1^OE^), and Par-1^RNAi^. (B) Quantification of nociceptive response to thermal stimulus of WT, Par-1 overexpression (Par-1^OE^) and Par-1 knockdown (Par-1^RNAi^) lines on vehicle media or AZT containing media. *F*(5, 85)=49.0, *P*=1.27E-23, one-way ANOVA. Post hoc Bonferroni (WT, WT AZT) *P*=2.61E-6, (Par-1^OE^, Par-1^OE^ AZT) *P*=0.353, (Par-1^RNAi^, Par-1^RNAi^ AZT) *P*=0.00577, (WT, Par-1^OE^) *P*=7.55E-6, (WT, Par-1^RNAi^) *P*=2.53E-5, (WT AZT, Par-1^OE^ AZT) *P*=0.0959, (WT AZT, Par-1^OE^ AZT) *P*=4.49E-9. (C) Quantification of number of terminal branches in the same genotypes as in B when exposed to AZT containing or vehicle containing food. *F*(5, 90)=206, *P*=1.02E-47, one-way ANOVA. Post hoc Bonferroni (WT, WT AZT) *P*=0.383, (Par-1^OE^, Par-1^OE^ AZT) *P*=0.0870, (Par-1^RNAi^, Par-1^RNAi^ AZT) *P*=0.000527, (WT, Par-1^OE^) *P*=2.98E-15, (WT, Par-1^RNAi^) *P*=2.76E-9, (WT AZT, Par-1^OE^ AZT) *P*=3.72E-18, (WT AZT, Par-1^OE^ AZT) *P*=1.53E-11. (D) Quantification of nociceptive response to mechanical stimulus of the same genotypes as in (B and C) when exposed to AZT containing or vehicle containing food. *F*(5, 62)=21.6, *P*=1.87E-12, one-way ANOVA. Post hoc Bonferroni (WT, WT AZT) *P*=8.39E-7, (Par-1^OE^, Par-1^OE^ AZT) *P*=0.0729, (Par-1^RNAi^, Par-1^RNAi^ AZT) *P*=0.00336, (WT, Par-1^OE^) *P*=6.16E-6, (WT, Par-1^RNAi^) *P*=0.676, (WT AZT, Par-1^OE^ AZT) *P*=0.404, (WT AZT, Par-1^OE^ AZT) *P*=0.0583. (E) Quantification of terminal branche fragmentation in the same genotypes as in (B–D) when exposed to AZT containing or vehicle containing food. *F*(5, 90)=184, *P*=1.05E-45, one-way ANOVA. Post hoc Bonferroni (WT, WT AZT) *P*=7.16E-16, (Par-1^OE^, Par-1^OE^ AZT) *P*=0.0780, (Par-1^RNAi^, Par-1^RNAi^ AZT) *P*=0.00265, (WT, Par-1^OE^) *P*=6.02E-22, (WT, Par-1^RNAi^) *P*=0.188, (WT AZT, Par-1^OE^ AZT) *P*=0.967, (WT AZT, Par-1^OE^ AZT) *P*=8.00E-17. WT=blue, Par-1^OE^=green, and Par-1^RNAi^ =red. Vehicle (−); AZT (+). **P<0.01, ***P<0.001, ****P<0.001; error bars=s.e.m., scale bar=50 µm; inset scale bar=20 µm.

**Fig. 8.**
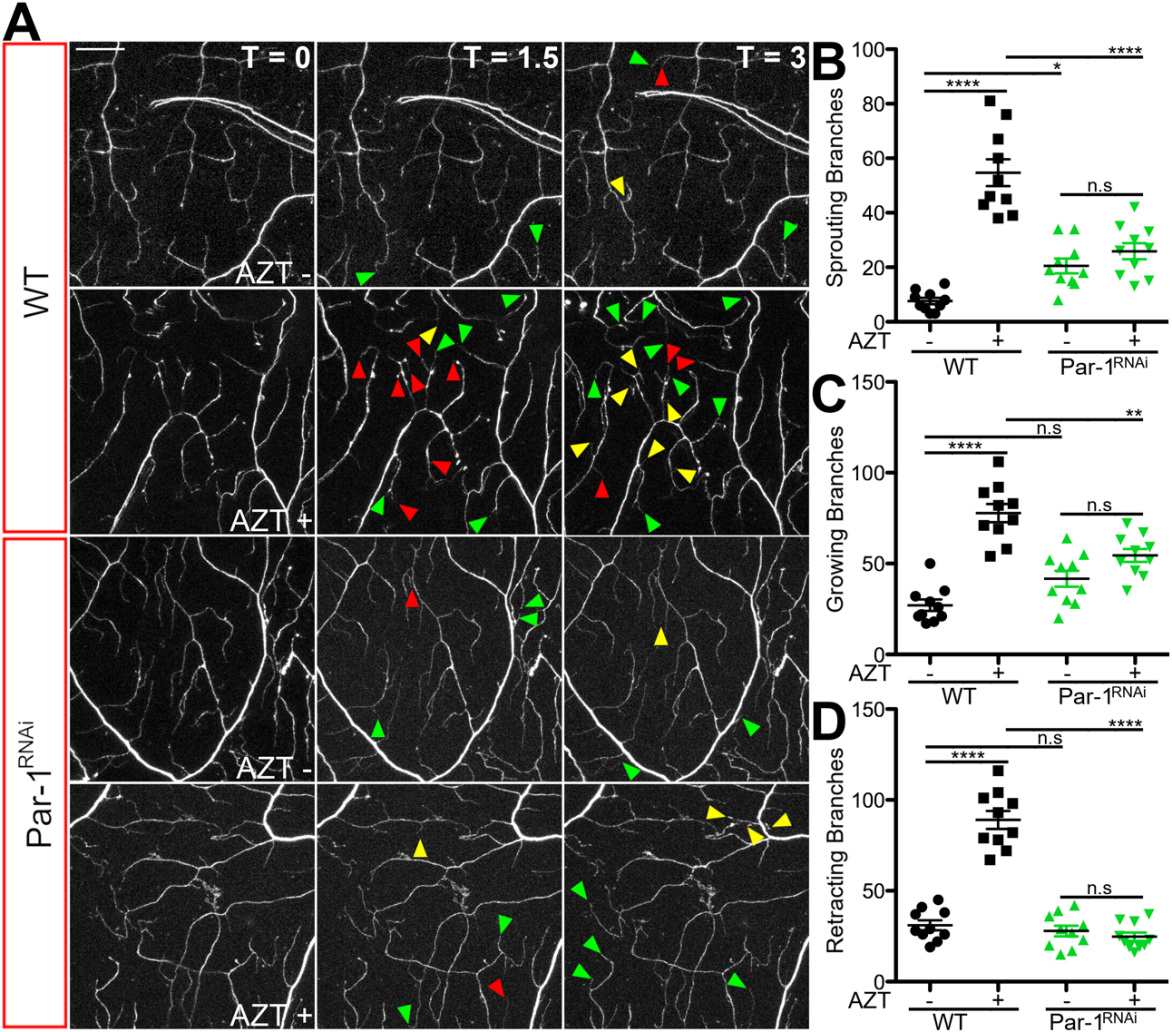
**Decrease in Par-1 also reduces the increased dendrite dynamics caused by NRTI.** (A) Representative images of GFP labeled C4da terminal dendrites imaged over a 3 h time period in larvae exposed to vehicle and AZT. The genotypes are indicated on the figure and are: WT, and Par-1^RNAi^. Time points shown are: 0, 1.5 h, and 3 h. (B) Quantification of sprouting branches of WT, and Par-1 knockdown (Par-1^RNAi^) lines raised in vehicle or AZT-containing food. *F*(3, 36)=38.1, *P*=2.80E-11, one-way ANOVA. Post hoc Bonferroni (WT, WT AZT) *P*=1.46E-5, (Par-1^RNAi^, Par-1^RNAi^ AZT) *P*=0.29, (WT, Par-1^RNAi^) *P*=0.0211, (WT AZT, Par-1^RNAi^ AZT) *P*=3.45E-5. (C) Quantification of growing branches of WT, and Par-1 knockdown (Par-1^RNAi^) lines raised in vehicle or AZT-containing food. *F*(3, 36)=27.4, *P*=2.12E-9, one-way ANOVA. Post hoc Bonferroni (WT, WT AZT) *P*=1.17E-5, (Par-1^RNAi^, Par-1^RNAi^ AZT) *P*=0.27, (WT, Par-1^RNAi^) *P*=0.0692, (WT AZT, Par-1^RNAi^ AZT) *P*=0.0014. (D) Quantification of retracting branches of WT, and Par-1 knockdown (Par-1^RNAi^) lines raised in vehicle or AZT-containing food. *F*(3, 36)=81.3, *P*=4.25E-16, one-way ANOVA. Post hoc Bonferroni (WT, WT AZT) *P*=8.98E-6, (Par-1^RNAi^, Par-1^RNAi^ AZT) *P*=0.40, (WT, Par-1^RNAi^) *P*=0.47, (WT AZT, Par-1^RNAi^ AZT) *P*=1.87E-6. WT=black, Par-1^RNAi^=green. Vehicle (−); AZT (+). *P<0.05, **P<0.01, ****P<0.0001; error bars=s.e.m., scale bar=20 µm.

Because time-lapse imaging revealed that terminal dendrites of neurons exposed to AZT were more dynamic, we wondered whether the instability of dendrites might contribute to their fragmentation-like phenotype. To test this hypothesis, we genetically manipulated the stability of dendrites by modulating the levels of Par-1 kinase, a microtubule-associated serine-threonine kinase whose levels are important in regulating dendritic stability during development (Herzmann et al., 2017). During normal development of dendrites, an increase in Par-1 kinase leads to a decreased stability of dendrites and vice versa. We found that increasing the levels of Par-1 kinase in the sensory neurons of WT larvae raised on AZT− food led to a significant decrease in the length of terminal dendritic branches, suggesting that they were unstable (Fig. 7), an idea that is consistent with a previous study (Herzmann et al., 2017). Decreasing the levels of Par-1 in larvae raised on AZT− food did not show any significant changes in dendrite morphology (Fig. 7). Finally, consistent with our hypothesis, Par-1 knockdown larvae raised on AZT had significant suppression of fragmentation-like phenotype of terminal dendrites (Fig. 7E). We also replicated these data using time-lapse imaging (Fig. 8). These data indicate that restoring the dendritic stability can suppress the fragmentation-like phenotype in flies raised on AZT.

Next, we asked whether restoring dendritic stability could also suppress the nociceptive hypersensitivity induced by AZT. To test this, we raised WT and WT larvae expressing Par-1^RNAi^ (Bayraktar et al., 2006; Iijima-Ando et al., 2012) specifically in their sensory neurons, on AZT and subjected them to both thermal and mechanical nociception paradigms. Indeed, we observed that restoring dendritic stability led to significant suppression of thermal nociceptive hypersensitivity (Fig. 7B). These data strongly suggest that alteration in dendritic stability may contribute to the fragmentation-like phenotype of the peripheral dendrites and this may possibly contribute to the nociceptive hypersensitivity in the *Drosophila* model of NRTI induced neurotoxicity.

## DISCUSSION

In this study, we show that *Drosophila* can be used as a model organism for investigating the mechanisms underlying peripheral sensory neuropathy and nociceptive hypersensitivity induced by NRTIs. Furthermore, our data suggest that NRTIs affect the stability of sensory neurons, which may make them susceptible to degeneration (Tan et al., 2011; Melemedjian and Price, 2012) and together, these may contribute toward the development of nociceptive hypersensitivity induced by the NRTIs in this model. However, the whether the fragmentation-like phenotype certainly underlies the nociception should still be investigate thoroughly. While this model is an important first step toward understanding the neurotoxicity of NRTIs, further work needs to be done to establish whether these changes are observed in vertebrate models, which we are currently investigating and unpublished data from our labs supports that conclusion (Bush, Tang, and Wairkar, unpublished data). Finally, further research on this topic is necessary because pain hypersensitivity has been linked to the development of chronic pain (Nielsen et al., 2009), a symptom prevalent in patients living with HIV (Cervia et al., 2010; Miaskowski et al., 2011; Merlin et al., 2013).

### *Drosophila* as a model to understand the mechanisms of NRTI-induced PSN

*Drosophila* has proved to be an excellent model in understanding the mechanisms of nociception (Lesch et al., 2010; Neely et al., 2010; Kim et al., 2012; Milinkeviciute et al., 2012). The characteristic “corkscrew” like behavior of *Drosophila* larvae to noxious stimuli has been exploited to screen for proteins that are required for nociception (Tracey et al., 2003). These initial studies have led the way to recent studies that have been instrumental in understanding the underlying mechanisms of peripheral neuropathy caused by the use of chemotherapeutic drugs such as Taxol (Paclitaxel) and vincristine (Bhattacharya et al., 2012; Brazill et al., 2018). Our study has furthered the use of *Drosophila* as a model to understand the mechanisms of nociceptive hypersensitivity induced by NRTIs, opening up the possibility of performing genome-wide forward genetic screens to elucidate the detailed mechanisms of NRTI induced neurotoxicity. Since many of the components for sensation, regulation, and integration of nociceptive signals are conserved from flies to vertebrates and humans (Im and Galko, 2012), this model may prove to be useful in shedding light onto the mechanisms of neurotoxicity induced by NRTIs. Finally, many signaling mechanisms underlying nociception are also conserved in *Drosophila* (Montell, 2001; Kim et al., 2012). Given the easy access to the peripheral nervous system (Corty et al., 2009) and the lower costs of performing unbiased forward genetic screens together with a plethora of genetic tools makes *Drosophila* a powerful model to investigate the mechanisms underlying neurotoxicity of NRTIs and possibly, by the other components of anti-retroviral drugs such as the protease inhibitors.

### Stability of dendrites and nociception

It is generally believed that activity patterns regulate the stability of synaptic communications. For example, during development, circuit refinement occurs based on activity (Kerschensteiner, 2014; Kutsarova et al., 2017) and active synapses are usually the ones that are stabilized. These connections can last for a long time, sometimes for decades in humans (Koleske, 2013). Indeed, loss of spine and dendrite stability has been observed in some psychiatric disorders and neurodegenerative disorders like Alzheimer's disease (Koleske, 2013). A recent study in a mouse model of Tauopathy also suggests that loss of synapse stability might precede the overt loss of neurons in these neurodegenerative diseases (Jackson et al., 2017). Consistent with these data, we found that exposure to NRTIs may also cause a loss of sensory neuron stability. Importantly, restoring the stability to the neurons led to the suppression of NRTI-induced sensory neuron fragmentation-like phenotype as well as nociception. While these data support the idea that synapse instability may underlie the PSN found in patients on chronic ART therapy, this idea needs to be rigorously tested in future studies to show beyond doubt that fragmentation-like phenotype and nociceptive hypersensitivity are indeed linked to each other. Also, more work needs to be done to understand the nature of this instability and the pathways that regulate it and ultimately, whether the fragmentation of sensory neurons is a possible mechanism that drives the hypersensitivity in pain underlying the use of ART and perhaps, in other chronic pain conditions. The combination of forward genetic screens in *Drosophila* and robust rodent pain models might offer an effective way to address these questions in the future.

### Chemotherapy-induced PSN versus NRTI-induced PSN

Chemotherapy-induced peripheral neuropathy (CIPN) is a significant problem associated with the use of chemotherapy drugs to treat cancer patients (Quasthoff and Hartung, 2002). The symptoms associated with CIPN present similar to that induced by NRTIs i.e., PSN. Recently, there has been a flurry of animal models of CIPN both in vertebrates and in flies (Authier et al., 2000, 2009; Bhattacharya et al., 2012; Benbow et al., 2016; Brazill et al., 2018). These studies on CIPN in model systems suggest that signaling mechanisms that play a vital role in axon degeneration (Bhattacharya et al., 2012; Brazill et al., 2018), which undergoes a characteristic degeneration associated with axonal injury (Coleman and Freeman, 2010), might also underlie CIPN. One of the common pathways associated with both axon degeneration cause by CIPN is the Di-Leucine zipper kinase (Dlk)-pathway. Reducing the levels of *dlk* (*wnd* in flies, Collins et al., 2006) results in the protection of distal axons in Wallerian degeneration models and, it also provides a similar level of protection for axons in *Drosophila* CIPN models (Bhattacharya et al., 2012; Fernandes et al., 2014; Brazill et al., 2018). However, our data show that the NRTI-induced fragmentation-like phenotype of peripheral sensory neurons is not suppressed by reducing the levels of *wnd* (Dlk), suggesting that the pathways that underlie CIPN and NRTI-induced fragmentation of dendrites may be different. It is important to note that these data are not without precedent because other recent studies also suggest that both regeneration and injury dependent degeneration of peripheral dendrites is independent of wnd/dlk pathway (Stone et al., 2014; Honjo and Tracey, 2018). Intriguingly, a recent model of CIPN that uses more optimized doses of Taxol suggests that dendrite stability may also play a role in the degeneration observed in CIPN models (Brazill et al., 2018). Together, these data suggest that even if there were a divergence in signaling pathways that mediate PSN, some of the mechanisms that lead to the sensory neuron degeneration might be common and may hinge on regulating the stability of peripheral sensory neurons.

## MATERIALS AND METHODS

### General methods

All the flies were cultured at room temperature. All the crosses were also performed at the room temperature. In all experiments involving nociception assays, adult *Drosophila* males and virgin females were transferred to food-containing vehicle, which usually was water, except DMSO that was used for experiments with pertaining to Taxol. Wandering third-instar larva were used for all experiments. All experiments within a figure panel were performed on the same day by the same experimenter and multiple repeats of the experiments were performed to achieve the significance value. Experiments where the expression in C4da neurons was used, the control flies were ppk-EGFP^5^ (Grueber et al., 2003). Otherwise, Canton S. was used as a control unless specifically stated. For each cross throughout the study, ∼10 virgin females were crossed to 7–8 males. These adult flies were allowed to lay eggs for 2–5 days per vial before being transferred to another vial. Therefore, in all experiments, the larvae developed in the food containing the drug or no drug.

### Thermal nociception assay

Wandering third instar larvae were placed in ∼1 ml of water in individual wells of a 96-well plate. If more than one genotype was being tested, they were placed opposite their drugged counterparts so that the wells were equidistant from the center of the block to ensure equal heat distribution. A black thermally conductive backplate was placed beneath the well plate for contrast so that the camera could track the larval movements with ease. A sensitive temperature probe was submerged in a centrally located unoccupied well with an identical volume of water. A camera was placed such that the wells with larvae and the temperature probe readout were clearly within the view of the camera. The recording of thermal nociception was started while the hot plate was at room temperature. The initial set point of the hot plate was room temperature and the temperature were raised in 0.1°C per 10 s increments. This rate was maintained throughout until the temperature reached 40°C. Videos were recorded for the entire period and the videos were quantified by two independent experimenters for the larval writhing response.

The nociceptive behavior used to qualify as nociceptive writhe is a well-documented corkscrew response (Im and Galko, 2012). The thermal nociception temperature at which each larva exhibited the corkscrew-like writhe response was recorded from the temperature of the probe inside the water bath. At 40°C, most of the larvae died and therefore, this last set of readings were not included in analyses. After analyzing the videos, three continuous corkscrew-like rolls were scored as nociceptive writhe. This temperature was designated as the minimum temperature required for the writhing response (Movie 1).

### Heat probe nociception assay and quantification

Heat probe nociception assay was performed as described previously (Chattopadhyay et al., 2012). Wandering third instar larvae raised at 22°C were collected and rinsed in PBS. Individual larvae were tested by touching the heat probe, consistently to the posterior third of the larvae. All the experiments were performed at the same time by the same experimenter. The larval response was recorded as non-responding, slow responding, and fast responding groups based on the following criteria: non-responders, larvae that did not exhibit nociceptive writhe within 20 s of the application of the thermal probe; slow-responders, larvae that exhibited nociceptive writhe between 5 and 20 s of the application of the thermal probe, and fast-responders, larvae that exhibited nociceptive writhe as soon as the probe was applied or within 5 s of the application of the thermal probe.

### Mechanical nociception

Von Frey filaments were designed such that they had similar diameter and flat tips. This protocol was adapted from Kim et al., 2012. These flat tips exert increasing pressure rather than force. The latter being the one used to test nociception in vertebrate animals. The filaments used in this paper were made in-house using a series of 12 calibrated monofilament fishing line segments (Berkley Trilene XL Monofilament Fishing Line), which were tethered to a hard-plastic handle. The filaments were calibrated by their ability to depress a balance consistently when vertically exerting a force until bent at roughly 30° from tip to tip. The series of 12 filaments consistently exuded following pressures: [100 kPa (0.50 mN), 150 kPa (0.75 mN), 200 kPa (1.01 mN), 250 kPa (1.26 mN), 300 kPa (2.36 mN), 500 kPa (3.93 mN), 700 kPa (5.50 mN), 1000 kPa (7.85 mN), 2000 kPa (15.71 mN), 3500 kPa (27.49 mN), 5000 kPa (39.27 mN)] over a 0.1 mm inserted tungsten wire tip. Experiments were performed beginning with the lowest pressure filament of 100 kPa (0.50 mN). Single larva was secured loosely in place with forceps and the filament tip was consistently pressed straight down on the lower third of the dorsal surface (preferably segment A6) of the larvae and released quickly. The larva was then released and free to move about, and the behavior was observed for 30 s for nociceptive writhe. If no noticeable nociceptive behavior was observed, the same larva was secured again and tested with the next filament in a sequence until a filament elicited the nociceptive behavior (writhe). We held the temperature of the test environment constant at 25–27°C because in our hands it affected the sensitivity of the larvae. Notably, temperatures below 24°C significantly decreased the larval response.

### Larval motility assay

Larval motility was assessed based on the method described in Nichols et al. (2012). Wandering third-instar larvae raised at 22°C were collected and rinsed in PBS. Individual larvae were assessed for their locomotor activity using a plastic-covered graph paper with 1 cm^2^ grid lines. Larvae were allowed to move freely for 1 min and the number of lines crossed was noted.

### *Drosophila* NRTI treatment

All flies were raised at room temperature (22°C) with natural day–night cycle. WT (*Canton S*) flies were placed in vials containing 2.5 ml of instant *Drosophila* media (‘blue food’, Carolina Biological Supply, Burlington, NC, USA) made with water base. NRTI experiments used water as a vehicle and the additional water in each NRTI vial amounts to less than 1 µl. NRTI dose response concentrations for AZT are: 0.026 μg/ml, 0.13 μg/ml, 0.26 μg/ml (main concentration used throughout), 1.3 μg/ml, 2.6 μg/ml, 13 μg/ml, 26 μg/ml, 52 μg/ml, 104 μg/ml, 208 μg/ml, and 416 μg/ml); or ddC: 0.0014 μg/ml, 0.014 μg/ml, 0.07 μg/ml, 0.14 μg/ml, 0.28 μg/ml, 0.56 μg/ml, 0.84 μg/ml, 1.12 μg/ml, 1.4 μg/ml, and 2.8 μg/ml. The AZT and ddC are water soluble and were stored in 1 mg/ml and 3.3 mg/ml concentrations respectively. AZT (Catalog number 3485) and ddC (Catalog number 220) were obtained from the NIH AIDS Reagent program (http://aidsreagent.org). Taxol experiments were treated with DMSO (Vehicle), with or without 30 μM of 1 mg/ml Taxol (Paclitaxel, Sigma, St. Louis, MO, USA) in DMSO. A stock of 1 mg/ml paclitaxel in DMSO was used for dilutions with identical amounts of DMSO used as control.

Ppk-EGFP^5^ virgin female flies (10) were crossed to males (7–8) required for the particular experiment in water-based food (control) and food treated with 0.26 μg/ml AZT diluted in similar amounts of water. Every cross in the study was made using at least 10 virgin females and 7–8 males. These adult flies were allowed to lay eggs for 3–5 days per vial. These crosses were maintained at room temperature (22°C). No significant differences were noted between males and female larvae or adult flies and their response towards the nociceptive stimuli, therefore, the data was collated for analyses.

### Dissection, imaging, and analyses

Specific imaging of C4da neurons was achieved via live imaging of ppk-EGFP^5^ lines that have been well characterized (Grueber et al., 2003). No immunostaining was used. All images were acquired using a Nikon Eclipse 90i laser scanning confocal microscope with either a 20× air or 60× oil objective. C4da neurons were visualized using the EGFP fluorescence of ppk-EGFP^5^. Each larva was mounted individually for imaging. Where the larvae were dissected, the dissections were performed in the following way: larvae were pinned at head and tail submerged in cold HL3 solution, an incision was made horizontally across the larval cuticle near the tail pin in such a way that only one side of the midline from pin to pin was cut, and a final incision was then made near the headpin. This method helped maintain total integrity of one-half of the Class IV da neurons (C4da), which was used for analyses. In our hands, the integrity of this one side (that was not cut) of the dendrites was maintained for as long as 15 min. In all cases, the staining appeared smooth and uniform throughout the C4da neurons. The trachea and guts were carefully removed using forceps and the larvae were unpinned and removed from the dissection plate to be placed cuticle side up on a glass slide. A small drop of cold HL3 was added to the larva on the slide before a small square coverslip was placed on top carefully to flatten and maintain the larval position for immediate imaging. The time taken from the first incision to the start of imaging was recorded and maintained within 2 min of variation; which for the entire experiment did not exceed 5 min (From the first cut to the imaging). Single C4da neuron and its dendritic field were imaged per larva from abdominal segment 3 or 4 with a laser dwell time of 1.68 µs per pixel at 1024×1024 spatial resolution. The sensory neuron was imaged through its entire z-dimension with a step size of 1 µm. The exception to this imaging method is the experiments in Fig. 5 where Canton S. and *dSARM*^−/−^ *Drosophila* lines were used. In this particular instance, the images shown are immunofluorescent-labeled neuronal membrane marker, HRP. Therefore, this figure does not show specific labeling of C4da neurons but instead labels all the sensory neurons. Apart from the fluorescence labeling, everything else was kept identical to the method described above.

For live imaging, larvae were rinsed in PBS and were directly mounted (without dissecting) within a coverslip cage in the HL3 solution. The coverslip cage was made to house the larva under slight pressure to hold it in place during the ∼3 h imaging period. The cage used in these experiments was made with two coverslips that touched the larvae such that the larval body was in between the coverslips. The dorsal projections of one C4da neuron per larva from abdominal segment 3 were imaged every 10 min for 3 h through the cuticle with a dwell time of 1.68 µs per pixel at 1024×1024 spatial resolution with a step size of 2 µm. Approximately, 10 µl of fresh HL3 was added to the coverslip cage every 10–15 min throughout the live imaging process to keep the larvae hydrated and to prevent hypoxia. The motility of larvae was assessed after the live imaging session was completed to assess their general health and only the images from larvae that showed normal motility were used in the analyses.

For analyses of dendrite fragmentation, confocal image stacks were converted to maximum intensity projections using ImageJ software. All analyses were performed on the entire C4da arbors of the single C4da neuron that was imaged. The total numbers of terminal dendritic branches and the total number of fragmented dendritic branches were counted manually, and the count was tracked using ImageJ. Discontinuous GFP pattern was analyzed manually and was counted as fragmented terminal dendrite. For analyzing the time-lapse images, confocal images in the time series were converted to maximum intensity projections using ImageJ software, and a random box of an area of 100×100 µm was drawn away from the cell bodies. Between each 10 min time point image, the total numbers of growing (from the same main branch), retracting, and sprouting (new branch formation) branches were counted manually for the entirety of the 3 h time lapse. The minimum length for qualifying as sprouting, growing, or retracting branch, the event had to be >1–2 µm in subsequently acquired images.

For determining muscle size, wandering third-instar larva were dissected and fixed with Bouin's fixative, which stains the muscles bright yellow. Bright-field images were taken, and ImageJ was used to quantify the muscle number 4 area of abdominal segment 3. At least ten larvae for each experiment and genotype were analyzed.

### Statistical analyses

All the experiments were performed with the experimenter blinded to the genotype of the larvae. The coded groups were then subjected to statistics before revealing their identity. Statistical analyses were performed using the GraphPad Prism software. The Student's *t*-test was used for comparison between two groups and one-way ANOVA followed by Bonferroni post hoc test was used when comparing multiple groups. Significance levels were set to 95% confidence intervals and the *P*-values are indicated in the respective figure legends.

## Supplementary Material

Supplementary information
