# Supplementary material for: Drosophila model of anti-retroviral therapy induced peripheral neuropathy and nociceptive hypersensitivity
Source: Biol Open. 2021 Jan 27;10(1):bio054635. doi: 10.1242/bio.054635 (PMC7860131; doi:10.1242/bio.054635)
Supplement: Supplementary information [file biolopen-10-054635-s1.pdf]

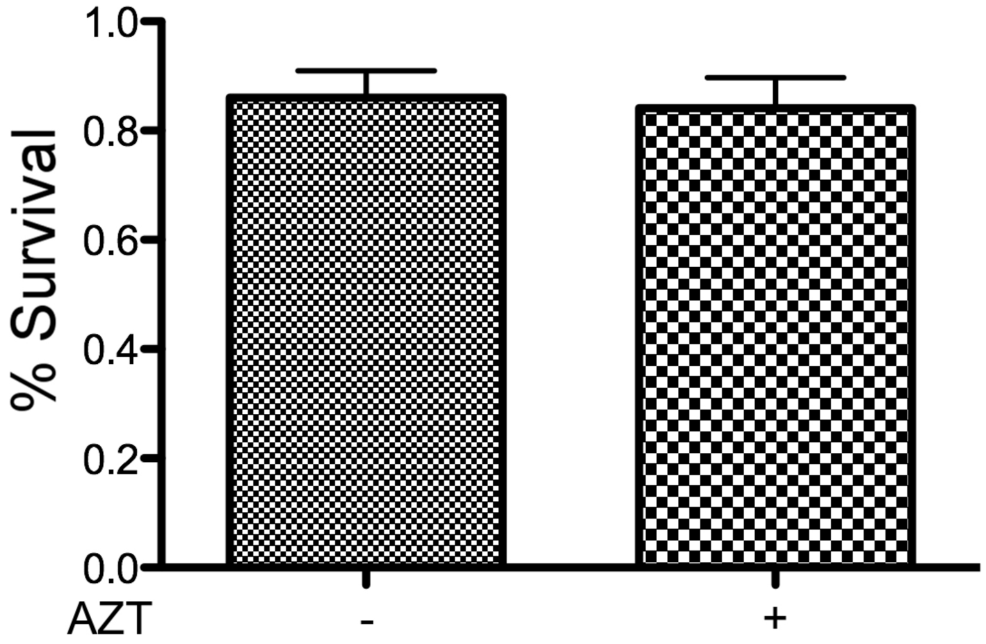

Figure S1: Effects of AZT on larval survival

Quantification of larval survival in vehicle and AZT food. 50 embryos of WT *Drosophila* were transferred to vehicle and AZT food. There was no significant difference in the number of larva and/or adult flies that survived. $p = 0.782$, t-test.

Biology Open • Supplementary information

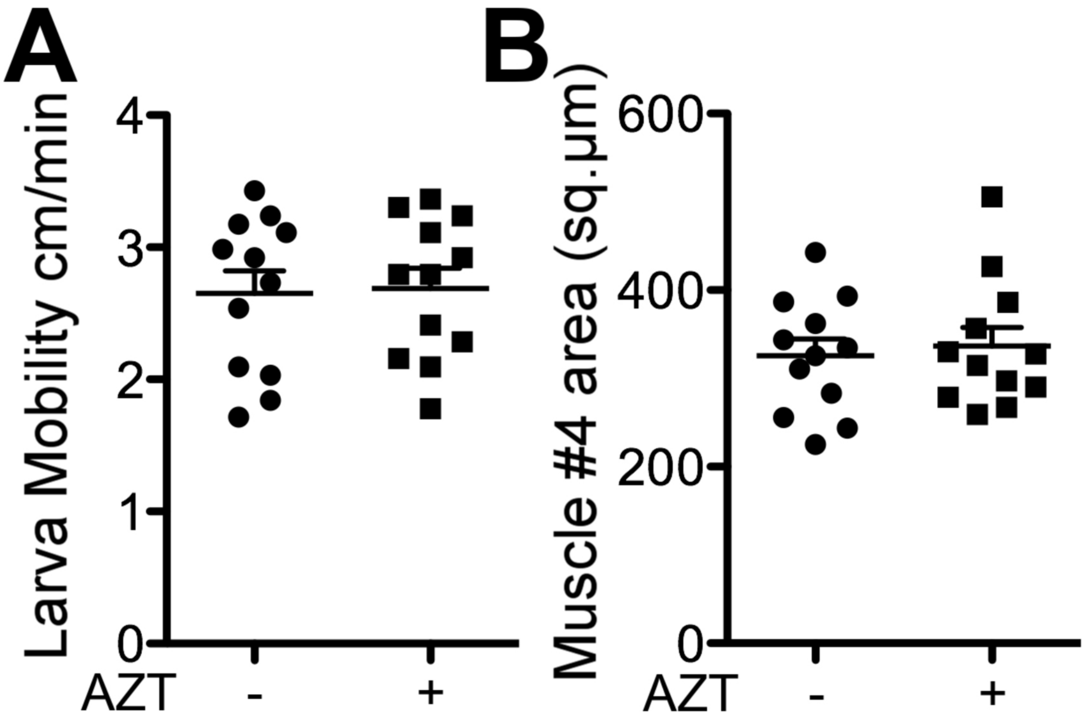

Figure S2: Larval motility and musculature are unaffected by AZT

A) Quantification of larval motility. There was no significant difference between larva raised in vehicle or AZT food. p = 0.873, t-test. **B**) Quantification of larva muscle 4 area (µm^2) from abdominal segment 3 and 4. There was no significant difference in the motility of larvae raised in vehicle or AZT food. p = 0.966, t-test.

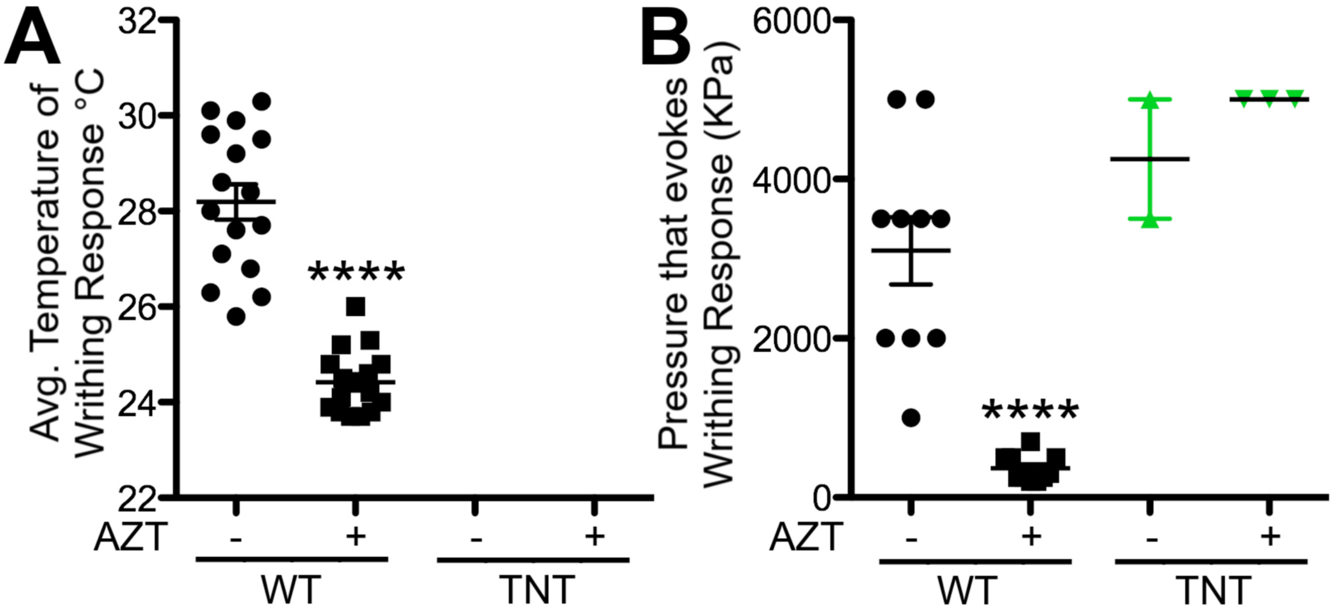

Figure S3: Nociceptive effects of NRTI are dependent on C4da sensory neurons

A) Quantification of temperatures required for the writhing response in WT larvae and larvae with ppk driven expression of tetanus toxin (TNT), grown in vehicle or AZT laced food. The larvae were non-responsive to changes in temperature when tetanus toxin light chain was driven in the C4da sensory neurons (last two bars). WT, AZT) p = 3.00E-9, t-test. There were 16 larvae used for each group. **B**) Quantification of mechanical stimulus required for nociception in the same groups. There were 10 larvae used for each group. Most TNT larva were non-responsive to all mechanical stimulus. WT, AZT) p = 5.79E-5, t-test. ****p < 0.0001; error bars = SEM.

Biology Open • Supplementary information

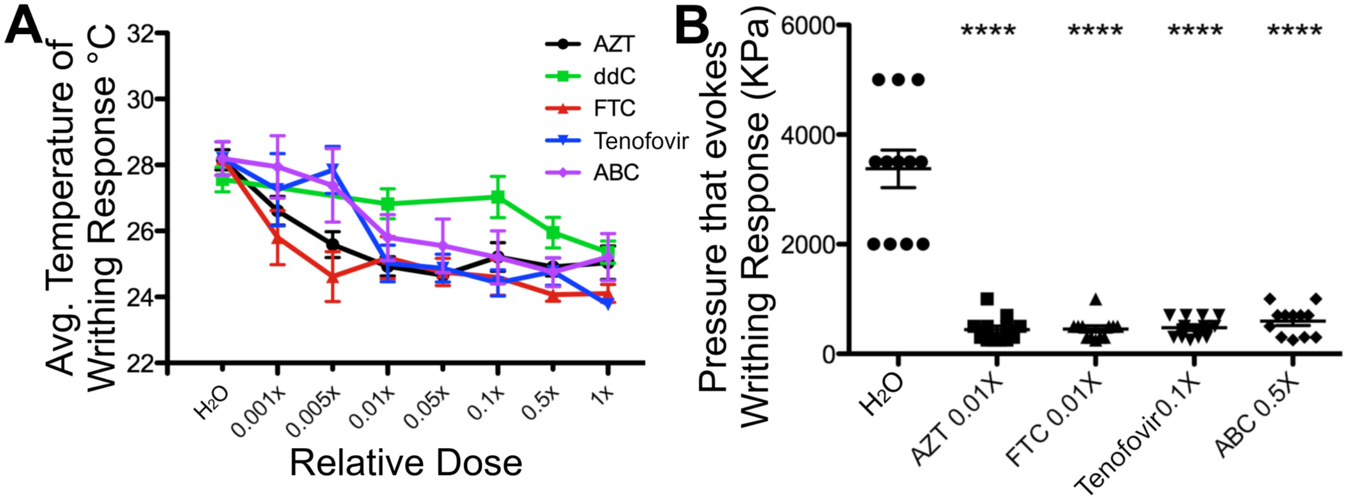

Figure S4: Newer NRTIs also induce thermal and nociceptive hypersensitivity in *Drosophila* model

A) Quantification of thermal response of larva to various relative doses of NRTIs. **B)** Quantification of mechanical hypersensitization of various NRTIs. $F(4, 55) = 62.0$, $p = 9.95E-20$, 1-way ANOVA. Posthoc Bonferroni (WT, AZT 0.01X) $p = 3.48E-6$, (WT, ABC 0.5X) $p = 5.38E-6$, (WT, FTC 0.01X) $p = 4.53E-6$, (WT, Tenovir 0.1X) $p = 3.75E-6$. ****$p < 0.0001$; error bars = S.E.M.

Biology Open • Supplementary information

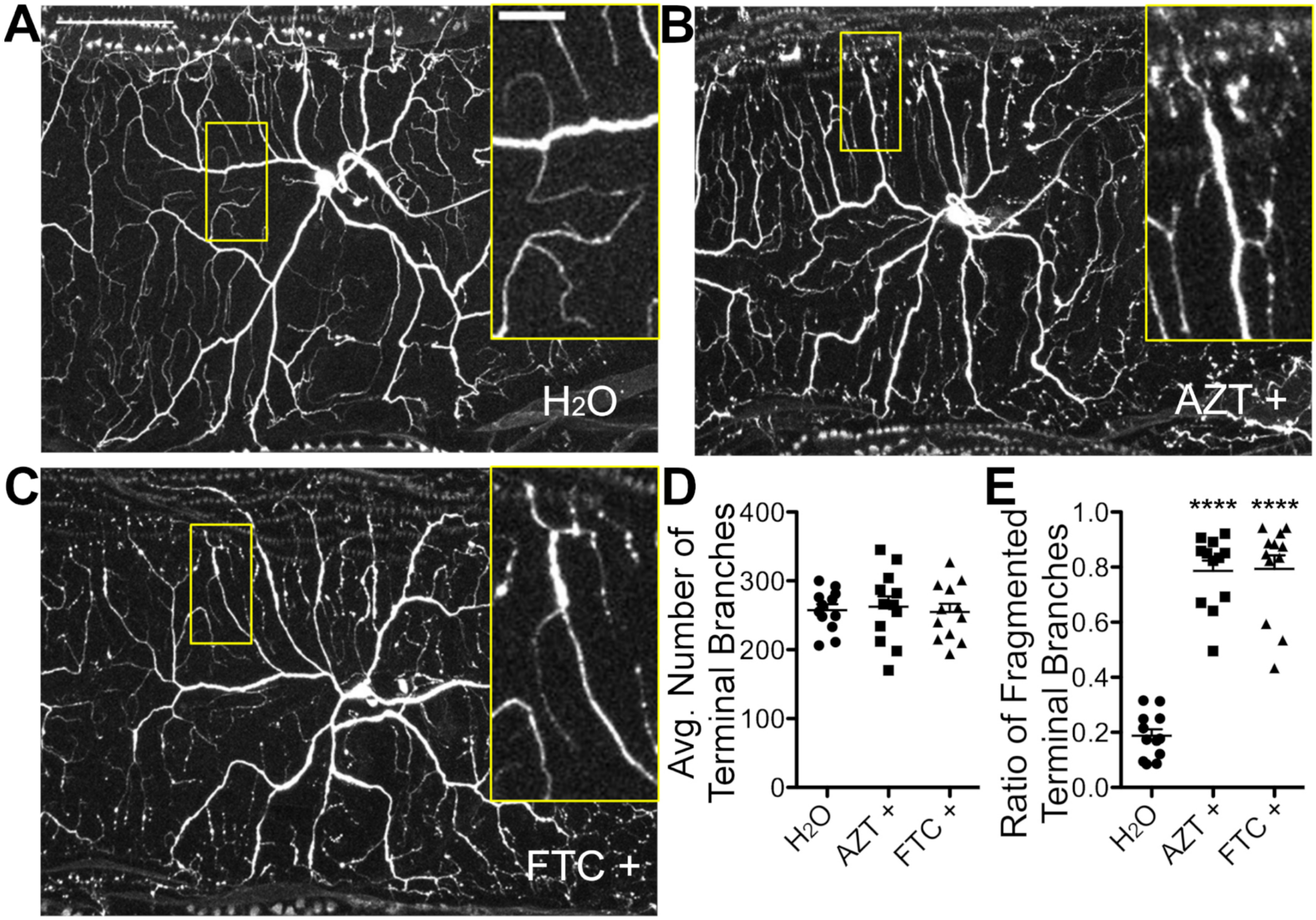

Figure S5: Exposure to FTC also leads to sensory neuron degeneration

A) Representative image of GFP labeled-C4da sensory neurons of third instar larvae raised on food containing vehicle(H_2O). **B)** Representative images of GFP labeled-C4da sensory neurons of third instar larvae raised on food containing AZT. **C)** Representative images of GFP labeled-C4da sensory neurons of third instar larvae raised on food containing FTC. **D)** Quantification of terminal branches of C4da sensory neurons in WT, AZT, and FTC larva. $F(2, 33) = 0.0983$, $p = 0.907$, 1-way ANOVA. **E)** Quantification of proportion of C4da terminal branches that exhibit fragmentation in WT, AZT, and FTC larva. $F(2, 33) = 81.2$, $p = 1.80E-13$, 1-way ANOVA. Posthoc Bonferroni (WT, AZT 0.01X) $p = 6.99E-8$, (WT, FTC 0.01X) $p = 5.75E-7$. ****$p < 0.0001$; error bars = S.E.M.; scale bar = 50 µm; inset scale bar = 20 µm.

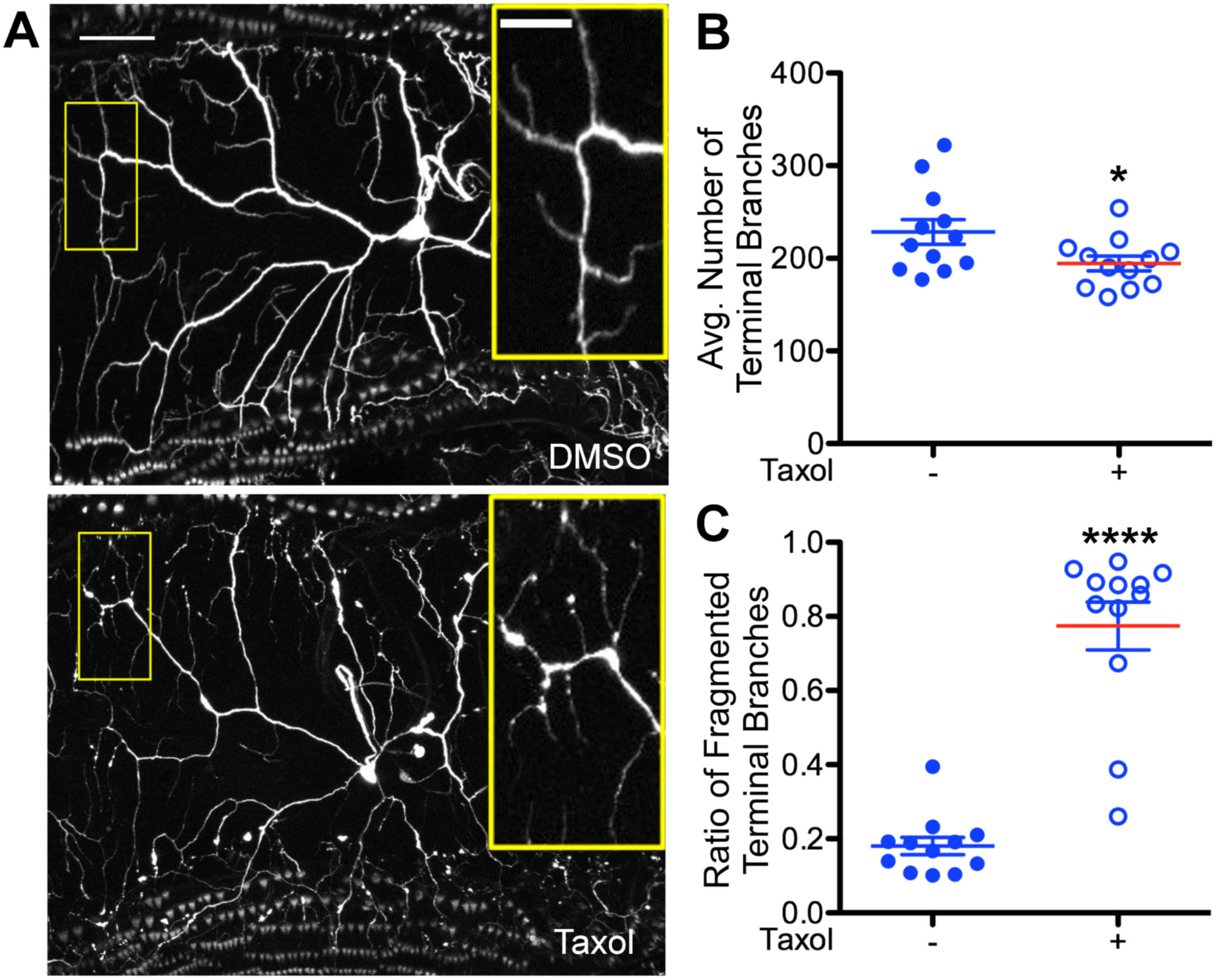

Figure S6: Taxol exposure also induces degeneration of sensory neurons.

A) Representative images of the C4da sensory neurons of third instar larvae comparing vehicle (DMSO) and Taxol dosed larvae. **B**) The number of terminal branches of C4da sensory neurons is decreased in larvae raised on Taxol. p = 0.0379, t-test. **C**) The proportion of C4da terminal branches that exhibit fragmentation is increased by exposure to Taxol. p = 1.53E-8, t-test. Vehicle -; Taxol +. N.S. = p > 0.05, ****p < 0.0001; error bars = SEM; scale bar = 50 µm; inset scale bar = 20 µm.

Biology Open • Supplementary information

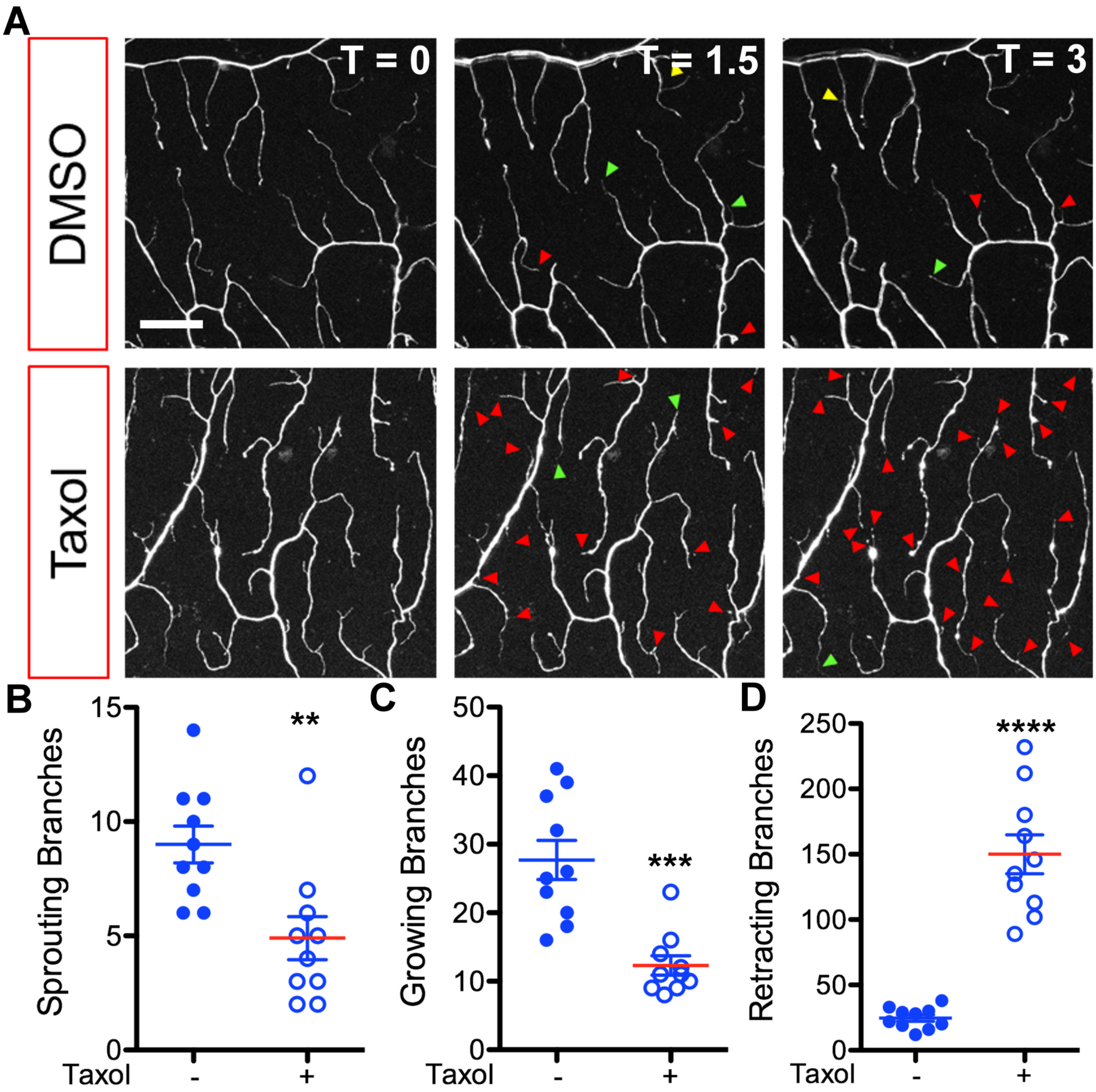

Figure S7: Taxol exposure induces increased dynamic changes in terminal dendrites of C4da sensory neurons.

A) Representative images comparing C4da dendrites over time in larvae exposed to vehicle (DMSO) or Taxol at time 0, 1.5 hours, and 3 hours. **B**, **C**) The number of dendrites exhibiting dynamic changes in the form of elongating ($p = 0.00398$, t-test), and sprouting ($p = 0.000132$, t-test) is decreased with exposure to Taxol while the quantity of retracting branches (**D**) is increased. $p = 1.40E-7$, t-test. Vehicle -; Taxol +. N.S. = $p > 0.05$, N.S. = $p > 0.05$, ***$p < 0.001$; error bars = SEM, scale bar = 20 µm.

Biology Open • Supplementary information

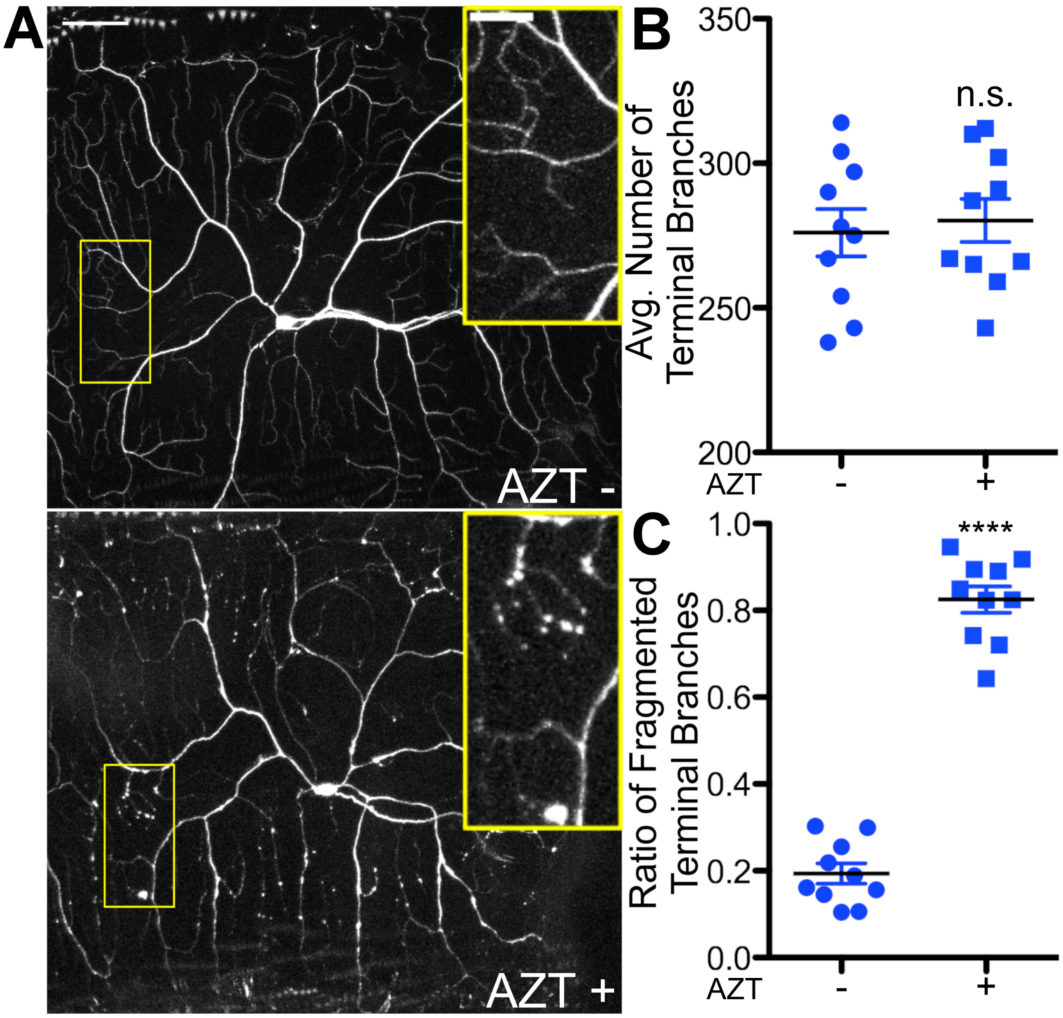

Figure S8: **Exposure to AZT leads to fragmentation of distal dendrites in live larva.**

A) Representative images of GFP labeled-C4da sensory neurons of third instar larvae raised on food containing vehicle or AZT. The images are taken through the cuticle of live larva. **B**) Quantification of number of terminal branches in live larval dendrites when exposed to AZT containing or vehicle containing food. p = 0.58, t-test. **C**) Quantification of terminal branch fragmentation in live larval dendrites when exposed to AZT containing or vehicle containing food. p = 1.85E-9, t-test. Vehicle (-); AZT (+). ****p < 0.001; error bars = SEM, scale bar = 50 µm; inset scale bar = 20 µm.

Biology Open • Supplementary information

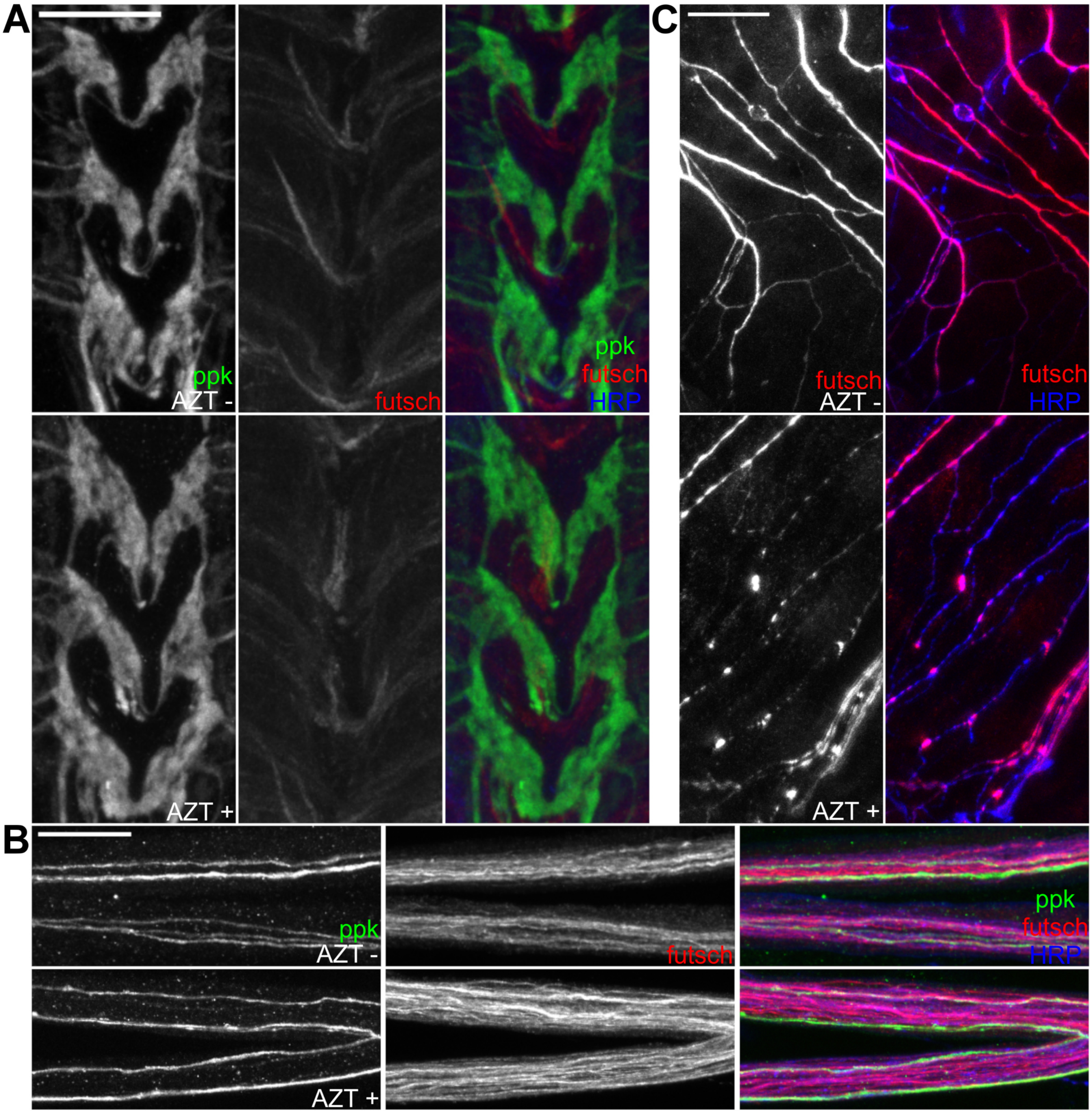

Figure S9: Futsch staining reveals similar fragmentation of terminal dendrite microtubules.

A) Representative images of VNCs of larva raised on food containing vehicle or AZT. Scale bar = 20 µm. **B**) Representative images of axons of larva raised on food containing vehicle or AZT. Scale bar = 20 µm. **C**) Representative images of terminal dendrites of larva raised on food containing vehicle or AZT. Scale bar = 20 µm.

Biology Open • Supplementary information

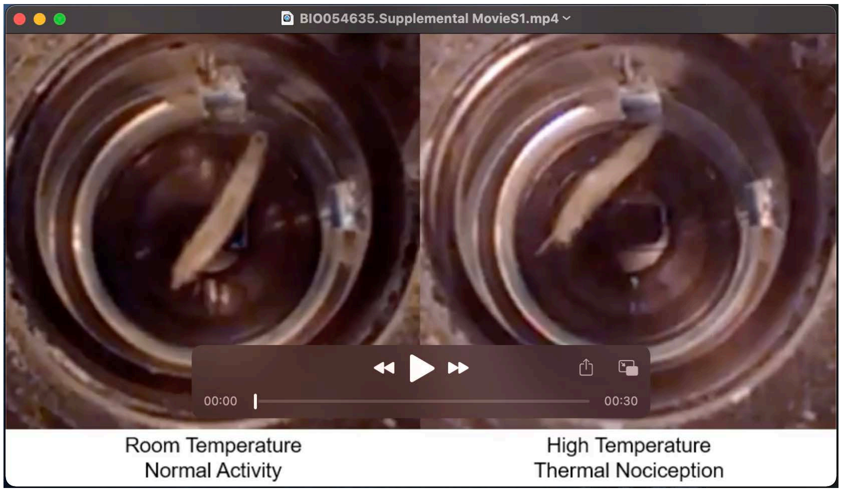

Movie 1: Representative larval behavior in thermal nociception assay.

Video demonstrating (idealized) larval behavior at sub-nociceptive temperature and characterized larval writhe at nociceptive temperatures.

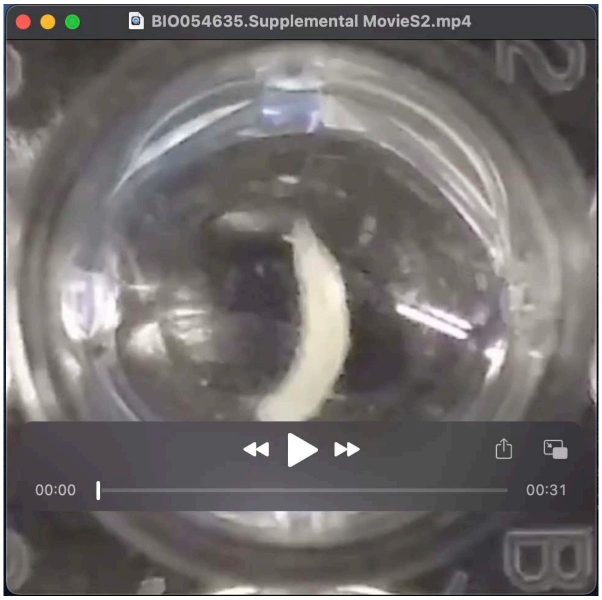

Movie 2: Representative larval behavior in thermal nociception assay.

Video of representative behavior of larva raised in AZT containing food in a room temperature water bath as utilized in thermal nociception experiments.

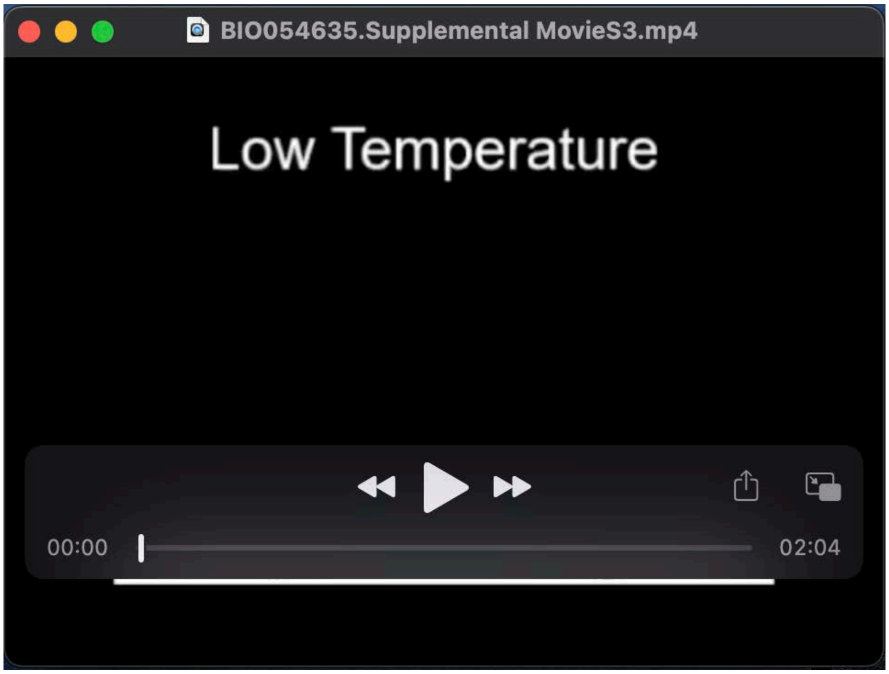

Movie 3: Representative larval behavior in thermal nociception assay.

Video shows behavior of larvae raised on normal (WT), and AZT containing food. Frames are time synced from the same video. The temperature frame includes the output of the thermal probe inserted into one of the wells filled with equal amounts of PBS as the one with larvae for accurate temperature measurement.

Biology Open • Supplementary information

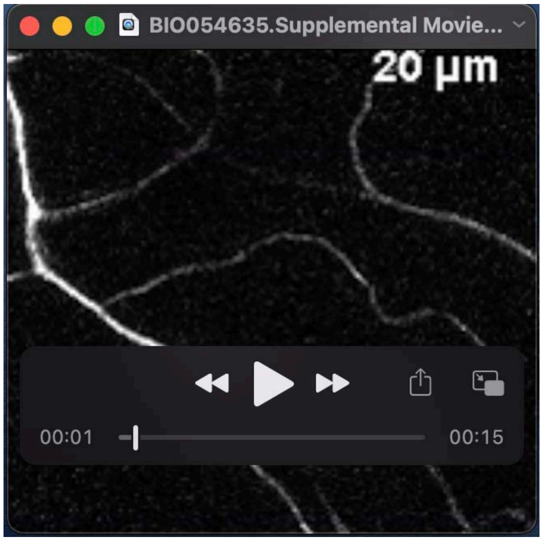

Movie 4

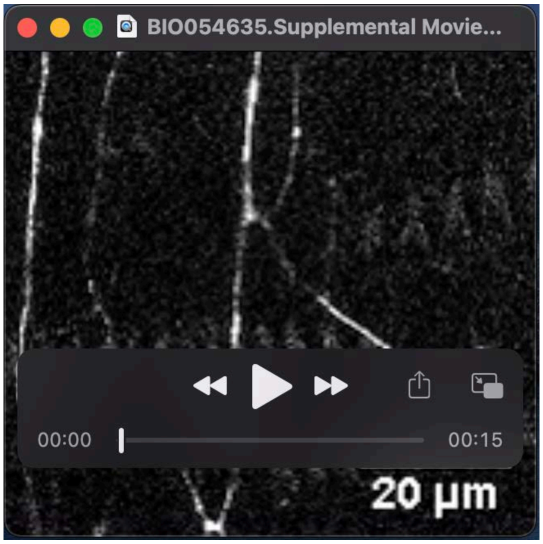

Movie 5

Movies 4 and 5: Example of live imaging of distal dendrites in WT and AZT exposed conditions.

Video showing 50µm by 50µm zoom of representative live imaging of larval terminal dendrites. Live imaging of larva grown in vehicle (**Movie 4**) or AZT (**Movie 5**) food show retraction (red arrowheads), elongation (Green arrowheads) and sprouting (Yellow arrowheads). Quantification of live imaging experiments is reported in figure 7.
